# Modification of tumor cell exosome content by transfection with wt-p53 and microRNA-125b expressing plasmid DNA and its effect on macrophage polarization

**DOI:** 10.1038/oncsis.2016.52

**Published:** 2016-08-08

**Authors:** M Trivedi, M Talekar, P Shah, Q Ouyang, M Amiji

**Affiliations:** 1 Department of Pharmaceutical Sciences, School of Pharmacy, Bouve College of Health Sciences, Northeastern University, Boston, MA, USA; 2 Faculty of Pharmacy, King Abdulaziz University, Jeddah, Saudi Arabia

## Abstract

Exosomes are responsible for intercellular communication between tumor cells and others in the tumor microenvironment. These microvesicles promote oncogensis and can support towards metastasis by promoting a pro-tumorogenic environment. Modifying the exosomal content and exosome delivery are emerging novel cancer therapies. However, the clinical translation is limited due to feasibility of isolating and delivery of treated exosomes as well as an associated immune response in patients. In this study, we provide proof-of-concept for a novel treatment approach for manipulating exosomal content by genetic transfection of tumor cells using dual-targeted hyaluronic acid-based nanoparticles. Following transfection with plasmid DNA encoding for wild-type p53 (wt-p53) and microRNA-125b (miR-125b), we evaluate the transgene expression in the SK-LU-1 cells and in the secreted exosomes. Furthermore, along with modulation of wt-p53 and miR-125b expression, we also show that the exosomes (i.e., wt-p53/exo, miR-125b/exo and combination/exo) have a reprogramed global miRNA profile. The miRNAs in the exosomes were mainly related to the activation of genes associated with apoptosis as well as p53 signaling. More importantly, these altered miRNA levels in the exosomes could mediate macrophage repolarization towards a more pro-inflammatory/antitumor M1 phenotype. However, further studies, especially *in vivo* studies, are warranted to assess the direct influence of such macrophage reprogramming on cancer cells and oncogenesis post-treatment. The current study provides a novel platform enabling the development of therapeutic strategies affecting not only the cancer cells but also the tumor microenvironment by utilizing the ‘bystander effect' through genetic transfer with secreted exosomes. Such modification could also support antitumor environment leading to decreased oncogenesis.

## Introduction

Exosomes are specialized membrane vesicles of about 30–150 nm in diameter and are shed in the extracellular environment by all living cells.^1^ Collecting these microvesicles shed in patient's serum and characterization of their contents is used as an early diagnostic marker for several diseases.^2, 3^ *In vitro* and *in vivo* studies indicate that the amount of exosomes shed by tumor cells is directly correlated with their invasiveness.^4, 5^ For example, vesicles shed spontaneously from highly metastatic B16 mouse melanoma (F0) cells when fused with weakly metastatic B16 mouse melanoma (F1) cells, the F1 cells could metastasize to the lungs.^6^ Similarly, exosome-mediated transfer of cellular cargo to neighboring cells has also been found to be affect tumor progression including angiogenesis, escape from immune response and extracellular matrix degradation.^7^ Generally the tumor cell shed exosomes facilitate transfer of soluble proteins,^8^ nucleic acids,^2^ functional transmembrane proteins,^9^ chemokine receptors,^10^ tissue factor^9^ and receptor tyrosine kinases such as epidermal growth factor receptor and human epidermal growth factor receptor 2.^11, 12^ Such exosome-based cargo thus promote oncogenic potential of tumor.^13^

Several non-coding RNA molecules such as microRNAs (miRs) are also present in these vesicles and can be transferred between neighboring tumor cells and other cells of the tumor microenvironment.^14^ The exosomal miRs are also used as a diagnostic marker in cancer.^14^ Specifically, studies in lung cancer have identified tumor-derived exosomes enriched in several different miRNAs including miR-21, miR-27 and miR-29-a, which may be potentially used as unique molecular markers to determine the specific cancer subtype.^15, 16^ Moreover targeting these miRNAs might also be a potential therapeutic modality for treatment of lung cancer.^17^ Functionally, these miRNAs are paracrine agonists for the Toll-like receptor family on the immune cells resulting in a Toll-like receptor-mediated pro-metastatic inflammatory response and can result in tumorigenesis and formation of secondary colonies at the metastatic site.^18^ For example, exosomes released by colon cancer cells contain mutated KRAS that can induce growth-promoting signals in the recipient non-transformed wild-type KRAS-expressing cells.^19^ It is noteworthy to mention that miRNAs contained in microvesicles released from tumor cells can also regulate their microenvironment when they are taken up by tumor-neighboring cells, including tumor-associated macrophages (TAMs) and dendritic cells.^3, 7^ Activation of these macrophages in the tumor microenvironment can be permissive of tumor progression and also induce metastasis.^8, 16^

Clinical evaluation has reported altered levels of several different miRNAs, including miR-21, miR-212, miR-7, miR-608, miR-198, etc. in non-small-cell lung cancer (NSCLC) patients^16^ and in A549 lung cancer cells,^17, 20, 21^ miRNAs associated with tumor migration, invasion and angiogenic properties have been identified.^20^ However, similar studies have not been performed on SK-LU-1 cells, which possess the KRAS G12D mutation and have mutated p53 expression. These cell lines are highly critical, especially since they have the features of clinically relevant tumors. While the clinical and preclinical application potential of exosomes is increasingly explored as a diagnostic marker for different types of cancers, the potential for exosome-based cancer therapy is also under investigation.^22, 23^ Several approaches have been investigated that include isolation of exosomes from serum samples of normal participant, transfecting them with the desired therapeutic moiety followed by re-introduction of these exosomes in cancer patients.^23^ However, despite the novelty of these approaches, issues such as immune activation and lack of target specificity related to the payload delivery have significantly hindered the clinical translation of exosomes-based cancer therapy.^1, 24^

In the current study, we provide a proof-of-concept for an alternative novel approach towards exosome-mediated reprogramming of the tumor microenvironment. Through non-viral transfection of KRAS/p53 mutant SK-LU-1 NSCLC cells with wt-p53 plasmid and hsa-miR-125b loaded in hyaluronic acid-poly(ethylene imine) and hyaluronic acid-poly(ethylene glycol) (HA-PEI/HA-PEG) blend nanoparticles, we try to indirectly modulate the payload in the exosomes secreted from these transfected SK-LU-1 cells. We also report the delivery of miR-125b for transfection to neighboring macrophages, which is known to induce macrophage activation from anti-inflammatory/pro-tumoral M2 phenotype to pro-inflammatory/antitumoral M1 phenotype. Such reprogramming of TAMs can induce an immunological activation against cancer at the site of tumor and result in ‘bystander effect' along with targeting cancer cells with wt-p53 as an apoptotic inducer. Thus, this approach has a synergistic activity as described previously by our group.^25^ Using Nanostring analyzer, we characterize the miRNA profile of exosomes, both before and after their treatment with wt-p53/miR-125b-encapsulated HA-PEI/HA-PEG nanoparticles. Lastly, we also characterized the functional consequences of such differential miRNA expression in exosomes by investigating the KEGG pathways associated with the significantly expressed miRNAs.

## Results

### Isolation and characterization of exosomes from SK-LU-1 lung adenocarcinoma cells

**Figure 1:**
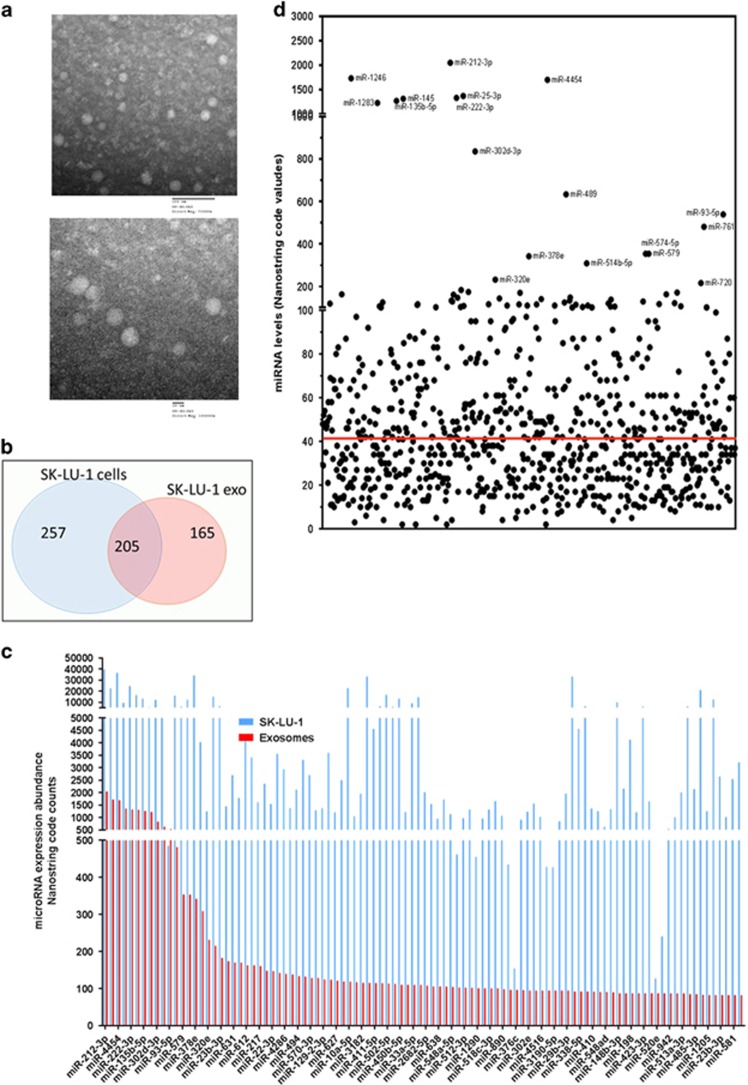
Characteristics of exosomes secreted from SK-LU-1 cells. (**a**) Transmission electron microscopy images of the exosomes isolated from non-transfected SK-LU-1 cells. The upper image was acquired at lower magnification, while the lower image is with higher magnification. (**b**,**c**) Venn diagram to illustrate miRNAs distribution between SK-LU-1 cells (SK/cells) and exosomes (SK/exo) secreted from non-transfected cells above the nonspecific background internal controls (red line in (**c**)). (**d**) Comparative distribution of miRNA expression profile in SK-LU-1 cells (SK/cells) and in exosomes (SK/exo).

Transmission electron micrographs revealed that the isolated exosomes showed a spherical morphology with an average size of 41 nm (Figure 1a; Supplementary Figure S1), as confirmed using a Malvern Nano-sizer (Malvern Instruments, Worcesteshire, UK). Consistent with these results, we also observed expression of CD63 marker in all of the different exosome samples using a western blot (Supplementary Figure S1).

### miRNA analysis in SK-LU-1 cells and exosomes

We identified and quantified the levels of common and specific miRNAs sequences expressed in the SK-LU-1 cells and their exosomes (Figures 1b–d). Utilizing RNA isolated from SK-LU-1 cells and their corresponding exosomes, we profiled the miRNAs. We found 462 known miRNAs, which were expressed in the cells significantly higher as compared with the proprietary nonspecific background internal control signal, whereas only 205 of these miRNAs were detected in the exosomes. Figure 1b shows this number of miRNAs shared between the SK-LU-1 cells and SK-LU-1-derived exosomes (SK/exo). Moreover, the cells contained 257 unique miRNAs that were not detected in the exosomes, whereas 165 exclusive miRNAs were detected only in the exosomes but were not significantly detected in the cells. It is also possible that certain miRNAs that were transferred into the exosomes with low levels retained within the cells. This phenomenon may result in missing miRNAs when the cDNA library in the course of human miRBase establishment was constructed. Supplementary Table S1 shows the top-ranking miRNA transcripts in the intracellular compartment as well as the exosomes.

As evident from Figures 1c and d, miR-212-3p, miR-1246, miR-4454, etc., were predominantly represented in the top-ranking miRNAs in both intracellular and exosome samples of SK-LU-1 cells. However, almost three quarters of the known miRNAs in the exosomes were detected at a significantly lower level as compared with their corresponding cells. But there were several miRNAs, which were present in the exosomes at a higher level as compared with the cells. Overall, these results show that the miRNA profiles in the cells and exosomes are notably different, which agree with the previous observations that miRNAs are sorted and released in exosomes.

### Target gene prediction and pathway mapping analysis

To evaluate the functions of exosome-loaded miRNAs in SK-LU-1 cells, we further chose the top 50 miRNAs in both: SK-LU-1 exosomes and cells. We next used DIANA mirPath together with DIANA-microT 4.0 and TargetScan 5.2 algorithms on miRNA targets associated with KEGG pathways. This allowed enrichment of biological pathways downstream of the 50 highly expressed miRNAs in exosomes, hence revealing the relationship between individual miRNAs and their influence on targeted genes.

Enrichment of genes involved in ‘cancer metastasis-related pathways' indicated possible involvement of ‘glycosiaminoglycan biosynthesis-chondroiton sulfate', ‘TGF-beta signaling pathways', ‘Wnt/signaling pathway', ‘Focal adhesion related pathway' as well as ‘ubiquitin mediated proteolysis pathway' (Table 1). This descending list of the top six cancer metastasis-related KEGG pathways based on a −ln(*P*-values) score threshold of ⩾2.00, which corresponded to lung cancer invasion properties as summarized in Table 1. We also performed hierarchical cluster analysis using DIANA miRPATH v.2.0 software (DIANA-Lab, Institute of Molecular Oncology, Vari, Greece) and associated the significantly expressed miRNAs along with their functional ontology from the KEGG pathway analysis (Supplementary Figure S2). Lastly, we also identified the target genes of the specific miRNAs associated with NSCLC from previous clinical diagnosis^16, 21^ (Supplementary Figure S3).

### Formation of plasmid DNA-loaded HA nanoparticles and transfection of SK-LU-1 cells with p53 and miR-125b either alone or in combination

**Figure 2:**
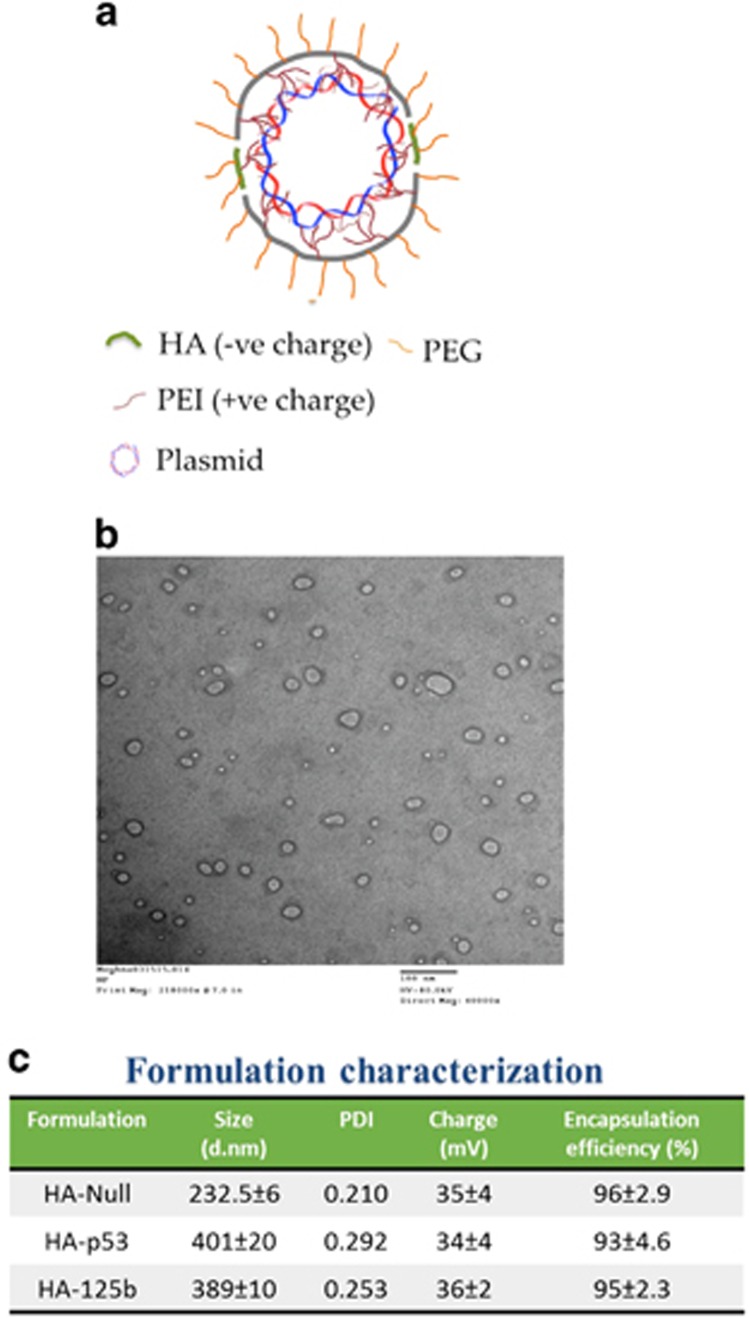
Characteristics of hyaluronic acid-poly(ethylene imine)/hyaluronic acid-poly(ethylene glycol) (HA-PEI/HA-PEG) nanoparticles. (**a**) Schematic representation of HA-PEI/PEG nanoassemblies encapsulated with plasmid DNA. (**b**) Transmission electron microscopy image of the self-assembling nanoparticles showing a spherical morphology. (**c**) Parting size and zeta potential (surface charge) analysis of the control and DNA-encapsulated nanoparticles. The hydrodynamic diameter of the particles was in range of 235-410 nm with a polydispersity index (PDI) of 0.2–0.3 and the surface charge was in the range of −33 to −38 mV.

**Figure 3:**
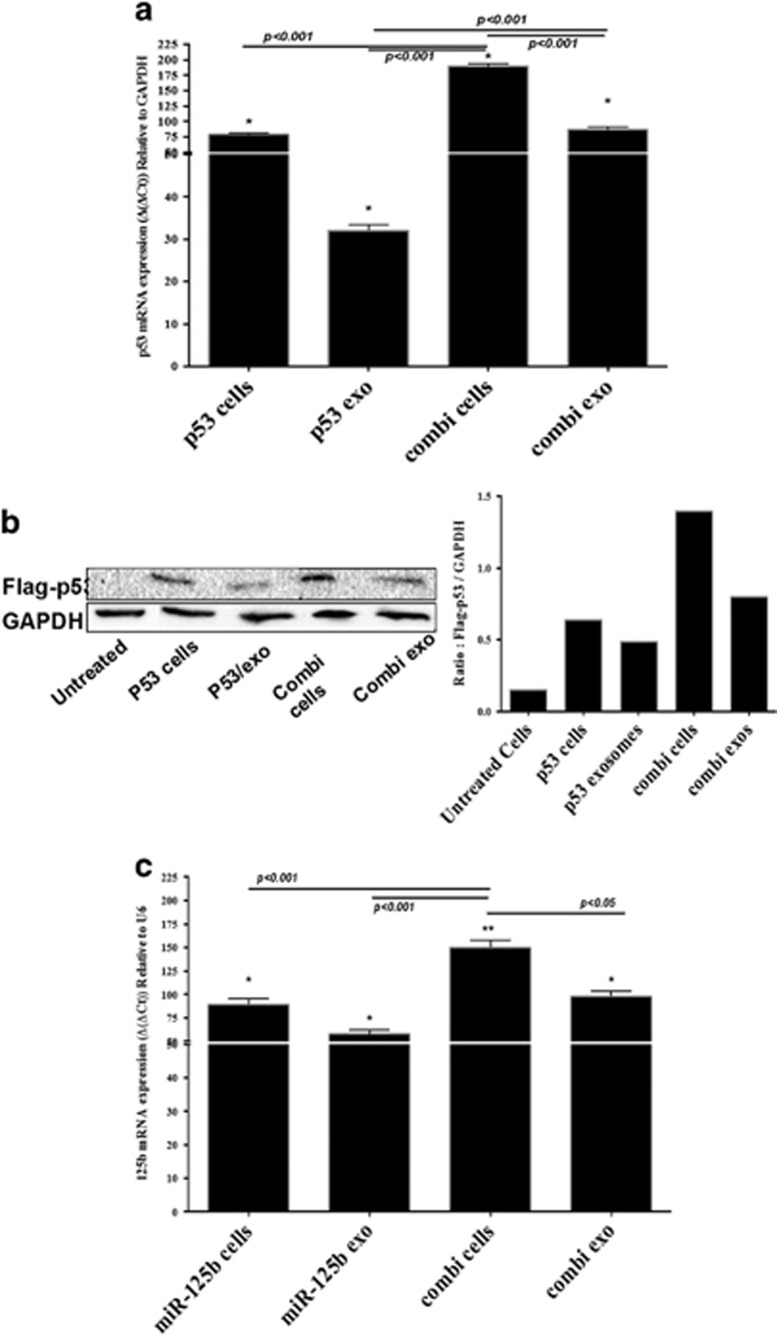
Evaluation of wt-p53 and microRNA-125b (miRNA-125b) transfection in SK-LU-1 lung adenocarcinoma cells and exosomes using plasmid DNA-encapsulated hyaluronic acid-poly(ethylene imine)/hyaluronic acid-poly(ethylene glycol) (HA-PEI/HA-PEG) nanoparticles. (**a**) Quantitative qRT–PCR analysis of expression of wt-p53 in cells (p53/cells) and in exosomes (p53/exo) when transfected with wt-p53 expressing plasmid DNA alone or in combination with miRNA-125b expressing plasmid DNA in cells (combi/cells) and in exosomes (combi/exo) after 18 h of incubation. (**b**) Qualitative and quantitative analysis of Flag-p53 protein expression using western blot in cells (p53/cells) and in exosomes (p53/exo) when transfected with wt-p53 expressing plasmid DNA alone or in combination with miRNA-125b expressing plasmid DNA in cells (combi/cells) and in exosomes (combi/exo) after 18 h of incubation. (**c**) Quantitative qRT–PCR analysis of expression of miR-125b expression in cells (miR-125b/cells) and in exosomes (miR-125b/exo) when transfected with miRNA-125b expressing plasmid DNA alone or in combination with wt-p53 expressing plasmid DNA in cells (combi/cells) and in exosomes (combi/exo) after 18 h of incubation. (*) *P*<0.05. Data represents mean±s.e.m., *n*=6.

Previously we have reported the development of self-assembling NPs with a spherical morphology and a size range of 200–400 nm^25^ and such transmission electron microscopy characterization was also performed in the current study (Figure 2b). We have also thoroughly characterized such nanoparticle assembly and reported the encapsulation efficiency of p53 and miR-125b plasmid^25^ and repeated such tests here as well (Figure 2c). We generated such nanoparticles and transfected SK-LU-1 cells with single or combination of these plasmids as previously reported.^25^ These nanoparticles have showed successful transfection of SK-LU-1 cells with wt-p53 and/or miR-125b plasmids. Here, we characterized and measured such plasmid gene expression not only in the SK-LU-1 cells but also in the secreted exosomes post-transfection of SK-LU-1 cells. As indicated in Figure 3a, the p53 mRNA levels in the exosomes were increased, irrespective of whether it was treated alone or in combination with miR-125b. However, the p53 mRNA levels in exosomes secreted from the SK-LU-1 cells treated with miR-125b in combination therapy (combi/exo) was much higher as compared with alone p53/exo. Similarly, the gene expression level of miR-125b was also elevated in the exosomes (Figure 3c). Additionally, the combi/exo had higher miR-125b levels as compared with 125b/exo. Lastly, the levels of p53 protein expression in the exosomes were also confirmed using western blot analysis and quantified using the ImageLab software (Bio-Rad, Hercules, CA, USA). Protein levels closely corresponded to the changes in the mRNA levels (Figure 3b). Thus, transfection of SK-LU-1 cells with nanoparticles encapsulating p53 and/or miR-125b can also be transferred to the exosomes.

### MiRNA content of exosomes after cellular transfection

As indicated above cells transfected with nanoparticles transfer their contents into exosomes. Next, we wanted to analyze if such expression induced any functional alterations in the secreted exosomal miRNA expression post transfection of SK-LU-1 cells with nanoparticles. We hypothesized that miRNA-125b or wt-p53 plasmid transfection might induce selective patterns of miRNAs in the exosomes, namely p53/exo, 125b/exo or combi/exo. For this purpose, the exosomes were collected post transfection with nanoparticles and RNA was isolated from these exosomes. miRNA expressions were analyzed by the Nanostring miRNA microarray platform same as with SK/exo and SK/cells. Differential patterns of expression were observed with each treatment of cells and their secreted exosomal miRNAs (Supplementary Figures S4). The top-ranking differential expression miRNAs are listed in Supplementary Tables S2–S4. Hierarchial clustering was also performed for each specific exosomal profile (p53/exo, 125b/exo and combi/exo) as depicted in Supplementary Figures S5–S7.

The miRNA profile differed not only between the treated SK-LU-1 cells and their exosomes, but also between different treatment groups. We compared the miRNAs secreted in different exosomes post transfection with nanoparticles (p53/exo, 125b/exo and combi/exo) to the miRNAs secreted in the SK/exo without any treatment. Compared with SK/exo, only 35 miRNAs were differentially expressed in the p53/exo (>2-fold changes). Out of these, 10 miRNAs were elevated whereas 25 miRNAs were downregulated. In contrast, 125b/exo had a total of 90 miRNAs differentially expressed (>2-fold change), wherein 42 miRNAs were downregulated and 48 miRNAs were elevated in expression as compared with SK/exo. Lastly, exosomes released from SK-LU-1 cells treated with combination of both plasmids; combi/exo had a high amount of changes in the miRNA expression, with a total of 81 miRNAs. About 64 miRNAs were upregulated and 17 miRNAs were downregulated in combi/exo as compared with SK/exo (>2-fold changes).

**Figure 4:**
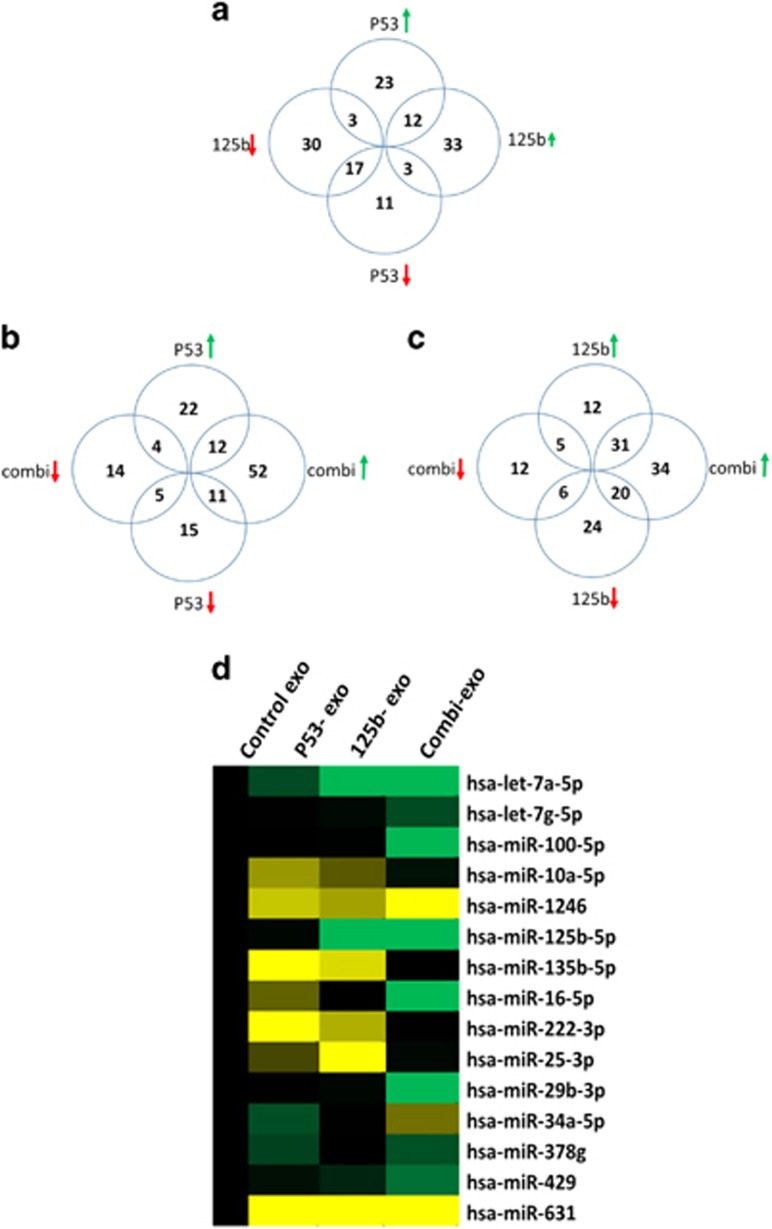
Comparison between differential microRNA (miRNAs) expression in different exosomes extracted from untreated and plasmid DNA-transfected SK-LU-1 cells. (**a**) Comparison between miRNAs secreted post-treatment of SK-LU-1 cells with individual plasmid expressing wt-p53 (wt-p53/exo) and miRNA-125b (miR-125b/exo). (**b**) Comparison between miRNAs secreted in exosomes post-treatment of SK-LU-1 cells with individual wt-p53 expressing plasmid (wt-p53/exo) and post-treatment with combination wt-p53 and miR-125b expressing plasmid DNA (combi/exo). (**c**) Comparison between miRNAs secreted post-treatment of SK-LU-1 cells with individual miR-125b expressing plasmid (miR-125b/exo) and post-treatment with combination miR-125b and wt-p53 expressing plasmid DNA (combi/exo). (**d**) Comparison of all miRNAs that were commonly expressed between all different treatment groups. About 15 miRNAs were significantly expressed in the exosomes from the transfected cells as compared with the control exosomes from non-transfected SK-LU-1 (SK/exo) groups (*P*<0.05).

Next, we analyzed if there were any commonalities in the induction of specific miRNA expression by different treatment regime (Figures 4a–c). About 12 miRNAs were upregulated in both p53/exo and 125b/exo treatments, whereas about 17 miRNAs were downregulated. Interestingly, there were three miRNAs which were downregulated in p53/exo but upregulated in 125b/exo, whereas there were three miRNAs, which were upregulated in p53/exo but downregulated in 125b/exo (Figure 4a). Similarly, when compared with combination treatment, 12 miRNAs were upregulated in both combi/exo and p53/exo, whereas about 31 miRNAs were upregulated in combie/exo and 125b/exo (Figures 4b and c). In contrast, five miRNAs were downregulated in common between combi/exo and p53/exo, whereas six miRNAs were downregulated in common between combi/exo and 125b/exo (Figures 4b and c). As such, the overall profiling results from the exosomes revealed a significant differential profile of miRNA expression post transfection with different nanoparticles. Specifically, treatment with combination of wt-p53 and miR-125b plasmid resulted in a slightly differential miRNA profile as compared qirh single plasmid treatment as well as from exosomes secreted by untreated SK-LU-1 cells (SK/exo).

### Functional analysis, target gene prediction and pathway mapping of exosomal miRNAs

To evaluate the functions of miRNAs loaded in exosome post transfection with nanoparticles, we further checked their potential targets through different computational algorithms using a web-based tool DIANA-microT-CDs along with the miRpath v2.0, same as used for the SK/exo miRNA profiling. Additionally, the Tarbase v7.0 was used to identify the putative gene targets using the KEGG pathway-based enrichment analysis. The comprehensive pathway analysis and the enriched miRNA sets as well as the putative gene targets are enlisted in specific Tables 2, 3, 4.

The global miRNA profile greatly differed in 125b/exo, p53/exo and combi/exo. Broad ranges of biological pathways and gene targets were identified to be associated with differentially expressed exosomal miRNAs. In specific, for miR-125b/exo, some functional pathways were related to exosome release (‘calcium signaling pathway', ‘ECM receptor interaction', ‘focal adhesion'). A lot of signaling pathway genes were related to cancer drug resistance (‘pathways in cancer', ‘MAPK signaling pathway', ‘PI3K/Akt signaling', ‘Transcriptional misregulation in cancer'). Interestingly, the ‘ubiquitin mediated proteolysis' pathway and ‘p53 signaling' pathway were also modified by the miRNAs secreted in 125b/exo. The cluster pathways analysis HeatMap is depicted in Supplementary Figure S8 and the relevant KEGG pathways are enlisted in Table 3.

Since in the p53/exo group, there were several miRNAs similar to the 125b/exo, the pathways affected did include signaling pathways in cancer drug resistance (‘PI3K/Akt signaling', ‘MAPK signaling', ‘Transcriptional misregulation in cancer') (Supplementary Figure S7). However, the percentage of miRNAs in this group was much higher as compared with 125b/exo. Similarly, the miRNAs related to exosome release (‘calcium signaling pathway', ‘ECM receptor interaction', ‘focal adhesion') were also higher in the p53/exo. More importantly, the pathways related to apoptotic signaling (‘HIF-1 signaling, ‘p53 signaling', ‘ubiquitin mediated proteolysis') were also elevated in the p53/exo. The specific pathways along with the corresponding gene target number are listed in Table 2.

In contrast to 125b/exo and p53/exo, the miRNAs from the combi/exo were associated with several different pathways. These miRNAs upregulated in combi/exo were associated with regulation of pathways related to macrophage functioning and T-cell receptor signaling and phagocytosis (sub groups: ‘cytokine-cytokine receptor interaction', ‘T-cell receptor signaling pathway', ‘Fc gamma R-mediated phagocytosis', ‘Notch signaling pathway', ‘NF-kappa B signaling pathway'), apoptosis-related pathway (subgroups: ‘Apoptosis', ‘p53 signaling', ‘Natural killer cell mediated cytotoxicity') (Supplementary Figure S7 and Table 4). However, similar to 125b/exo and p53/exo, the miRNAs downregulated in combi/exo were associated with signaling pathways in cancer (‘transcriptional misregulation in cancer', ‘MAPK signaling', ‘PI3K-Akt signaling', ‘VEGF signaling'). We also performed a comparative diagram for depicting the similarities and differences between the different exosome groups. As indicated several pathways were shared by these groups, mainly related to the cancer cell and p53 signaling; however the combi/exo group definitely stood out to be slightly different in form of its ability to activate the cytokine and chemokine signaling (Supplementary Figure S9). Thus, the HA nanoparticles treatment with combination approach of p53 and miR-125b altered the global miRNAnome profile in the exosomes secreted from SK-LU-1 cancer cells (Supplementary Figure S4 and Table 4).

It is noteworthy to mention that we have previously quantified the transfection efficiency of our nanoparticles and validated it against commercial standard lipofectamine 3000 reagent.^25^ And hence, we did not undertake such experiments in the current studies. However, to ensure that the miRNA secretion in the exosomes was specific to the plasmid treatment and not the HA nanoparticles itself, we did measure the changes in specific miRNA levels in exosomes following treatment with blank nanoparticles, naked plasmids using specific Taqman qPCR probe sets (Supplementary Figure S8).

### Exosomal miRNAs transfer to macrophages

**Figure 5:**
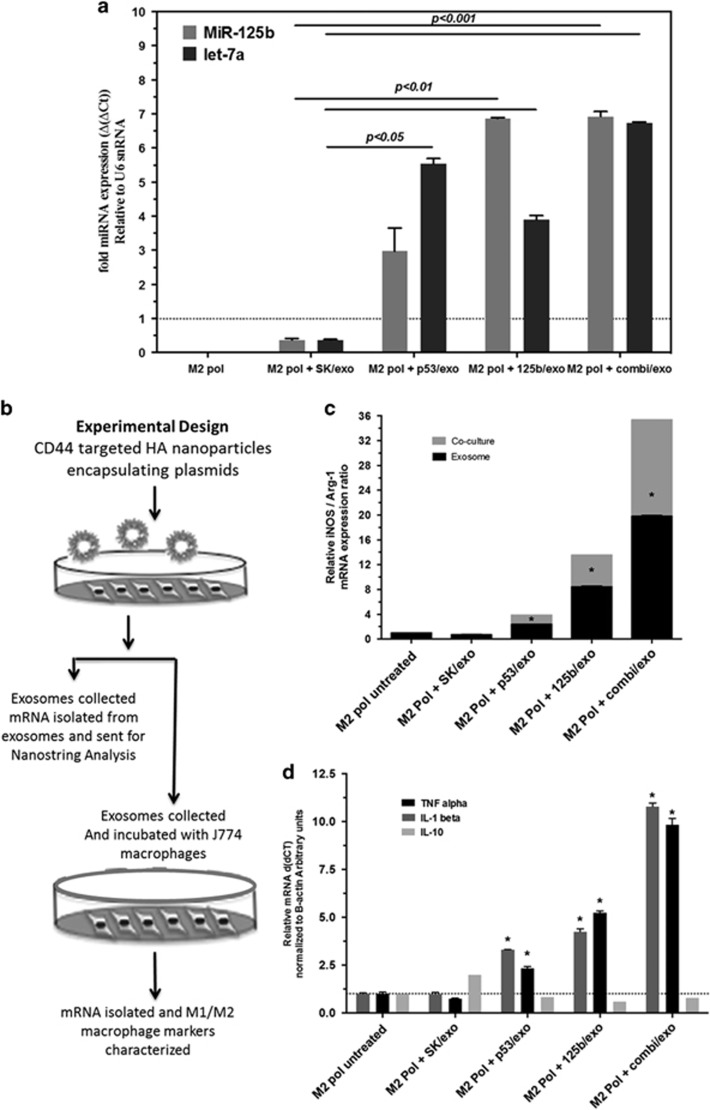
Macrophage repolarization with exosomes from wt-p53 and miRNA-125b transfected SK-LU-1 cells. (**a**) qRT–PCR analysis following exosome-induced changes in the miR-125b expression profile of J774.A1 murine macrophages. The J774.A1 cells pre-polarized to M2 phenotype using IL-4 were treated with exosomes from non-transfected SK-LU-1 cells (SK/exo) and exosomes obtained after transfection with plasmid DNA expression wt-p53 (p53/exo), miRNA-125b (miR-125b/exo) and combination wt-p53/miR-125b (combi/exo)-transfected SK-LU-1 cells after 18 h. (**b**) Schematic of the experimental design for exosome-mediated changes in the iNOS/Arg-1 gene expression ratio as well as pro- (tumor necrosis factor (TNF)-α and IL-1β) and anti-inflammatory (IL-10) cytokine expression in J774.A1 macrophages after treatment with exosomes from non-transfected and transfected SK-LU-1 cells. (**c**) The expression ratio of iNOS/Arg1 mRNA in J774.A1 macrophages after treatment with exosomes from non-transfected SK-LU-1 cells (SK/exo) and exosomes obtained after transfection with plasmid DNA expression wt-p53 (p53/exo), miRNA-125b (miR-125b/exo) and combination wt-p53/miR-125b (combi/exo)-transfected SK-LU-1 cells after 18 h. (**d**) The expression of pro- (TNF-α and IL-1β) and anti-inflammatory (IL-10) cytokine expression in J774.A1 macrophages after treatment with exosomes from non-transfected SK-LU-1 cells (SK/exo) and exosomes obtained after transfection with plasmid DNA expression wt-p53 (p53/exo), miRNA-125b (miR-125b/exo) and combination wt-p53/miR-125b (combi/exo)-transfected SK-LU-1 cells after 18 h. For qRT–PCR analysis, all of the individual gene expression markers were normalized to beta-actin and untreated control samples using relative quantification by delta(delta(Ct) method. * indicates comparison against control designated as 1. One-way ANOVA followed by *post hoc t*-test with multiple comparisons, *P<0.05. Data represent as mean±s.e.m., *n*=6.

The presence of selective miRNA patterns in combi/exo opens up the intriguing possibility that these miRNAs may propagate their signals and transfer the message to their neighboring cells via exosomes. Since macrophages make up a large population of tumor cells, we tested this hypothesis by incubating J774 macrophages with combi/exo and compared the levels of specific miRNAs found in J774 macrophages with those of untreated cells. We performed qRT–PCR analysis, which showed that combi/exo-treated J774 macrophages carried elevated levels of miR-125b and let-7a as compared with untreated J774 macrophages (normalized to U6 snRNA; Figure 5a). These elevated levels of miRNAs present at significantly higher levels in J774 macrophages cells relative to the untreated cells suggests transfer from exosomes. This is not surprising, especially since the co-culture of SK-LU-1 cells post transfection with combination therapy and the J774 macrophages cells resulted in significantly higher expressions of miR-125b and p53 as compared with untreated cells (previously published^25^).

### Repolarization of macrophages following incubation with exosomes

The M2-polarized TAMs can be characterized by an increase in the iNOS/Arg1 expression ratio.^26^ The inducible nitric oxide synthase (iNOS) is tightly correlated with M1 phenotype during inflammation, whereas arginase-1 (Arg1) is generally involved with anti-inflammatory role in the M2 phenotype. Arg1 uses the same substrate as iNOS and hence only one is preferably synthesized in the macrophages, thus allowing iNOS/Arg1 to be a good marker of the repolarization of macrophages. The TAMs are generally heterogeneous, although a majority of them were of the M2 anti-inflammatory/pro-tumoral phenotype. The M2 polarization markers can be achieved *in vitro* by treatment of macrophages with interleukin-4 (IL-4).^26^ We recently reported that co-culturing the M2-polarized J774 cells with SK-LU-1 cells transfected with nanoparticles coding for miR-125b in combination with p53 plasmid showed an ~35 fold increase in the iNOS/Arg1 ratio in J774 macrophages (*P*<0.05, relative to non-transfected but M2-activated cells).^25^ They also induce a pro-inflammatory microenvironment as supported by the elevated mRNA levels of other cytokines and interleukins, for example, tumor necrosis factor-α, IL-1β, IL-6 showed a similar elevation. We wanted to investigate if such changes were mediated by secreted exosomes. Hence, we treated SK-LU-1 cells with nanoparticles encapsulated with plasmids coding for p53 alone or in combination with miR-125b and collected the exosomes (Figure 5b). Following the same paradigm we treated M2-polarized J774 macrophages with p53/exo, 125b/exo or combi/exo and measured the iNOs/Arg1 ratios. As indicated in Figure 5c, the iNOS/Arg1 ratio was elevated, indicative of M1 macrophage phenotype post-treatment with all the three different types of exosomes. Corresponding changes were also observed in other cytokines indicative of an M1 phenotype (Figure 5d). However, only treatment with 125b/exo and combi/exo depicted a significant elevation as compared with untreated cells (*P*<0.05). The treatment with combi/exo resulted in ~25-fold elevation in the iNOS/Arg1 ratio in the J774 macrophages. Several other relevant macrophage markers, namely IL-β, IL-6 and tumor necrosis factor-α, were also measured and supported the iNOS/Arg1 ratio and an induction of a pro-inflammatory state (Figure 5d).

## Discussion

As a preliminary proof-of concept, our current study shows that transfection of tumor cells with plasmid using the targeted drug-delivery approach can modify the miRNA content of exosomes released from these cells. In specific, we report that transfecting SK-LU-1 lung adenocarcinoma cells with miR-125b and/or wt-p53 plasmid loaded in HA-PEI/HA-PEG nanoparticles, we can overexpress these genes not only in the SK-LU-1 cells but also in the secreted exosomes. Thus, this approach can potentially reprogram the exosome composition without the need for their purification. It is important to note that the current study characterized the exosomal and relevant miRNA changes only in *in vitro* studies. Although *in vivo* experiments were not performed in the current study, such *in vivo* manipulation of the exosome content can lead to broader dissemination of the delivered therapeutic molecules via bystander effect to distant parts within the tumor, thereby potentially affecting other tumor cells as well as other cells harbored within the TME. Such studies in animal models of patient-derived xenograft will be performed in the future. Furthermore, the current studies also support our previous results for the use of non-viral drug-delivery system in form of HA-PI/HA-PEG to transfect the SK-LU-1 cells as well as their exosomes.

miRNAs can regulate gene expression, controlling many cellular functions such as proliferation, differentiation, apoptosis, oncogenesis and drug sensitivity in tumor cells.^27, 28^ miRNAs are known to undergo genetic alterations, such as amplification, deletion and epigenetic silencing, which can ultimately activate oncogenes and inactivate tumor suppressors in cancer cells.^27, 28^ Certain miRNAs are consistently dysregulated across many cancers, including NSCLC.^16, 21^ In the current study, we have observed that exosomal miRNAs associated with antiapoptotic pathways, or several different signaling pathways in cancer, were decreased in the combi/exo group, whereas the levels of pro-inflammatory and pro-apoptotic miRNAs were elevated. Moreover, these changes were more prominent with the combination therapy as compared with either plasmid alone. This is indicative of a potential synergistic action between the two plasmids, namely wt-p53 and miR-125b. Furthermore, they are also supportive of our previous results, wherein we observed an elevated *in vitro* and *in vivo* anti-tumor efficacy of a combination approach of combination of p53 and miR-125b in *in vitro* and *in vivo* studies with Kras G12D cell lines and animals.^25^

Tumor microenvironment is abundantly populated with immune cells, including macrophages that contribute to the growth and development of the disease. Role of miRNAs in regulating the balance between the M1 and M2 phenotypes of macrophages as well as impacting the recruitment of other immune cells in the tumor microenvironment is well established.^16, 26, 29^ Even recent studies from our group reported that that treatment with combination therapy of miR-125b and p53 encoding plasmid results in elevated M1 macrophages both *in vitro* and *in vivo*.^25^ In the current study, we also observed that combi/exo treatment of J774 macrophages induced repolarization towards M1 phenotype with elevation in pro-inflammatory cytokines. This was in addition to the fact that several miRNAs associated with pathways like ‘T-cell activity' and ‘natural killer cell activity' were also upregulated in combi/exo. Thus, nanoparticles could not only alter the global exosomal miRNA profile but subsequently also induce exosome-based TAM repolarization towards a pro-inflammatory stage.

Most clinical studies suggest that NSCLC with TP53 alterations carries worse prognosis and may be relatively more resistant to chemotherapy and radiation.^30^ Chemoresistance is a major stumbling block for treatment with current chemotherapeutic modalities leading to relapse and elevated oncogenic potential.^31^ Exosomes shed by the tumor cells have long been confirmed as cell–cell communicators that can transfer messages from one cell to another and can play a vital role in development of the disease, including exosome-mediated resistance and metastatic transmission.^1^ Some of the miRNAs that were downreglated in the combi/exo group were associated with functional pathways related to chemoresistance and drug sensitivity. Hence, our drug-delivery approach might also be used in conjunction to chemotherapeutic drugs, which could potentially result in elevated efficacy and potency of chemotherapeutic drugs. These results also explain our previously published results, wherein we observed increased sensitivity of SK-LU-1 cells as well as KP mouse model (Kras G12D mutation carrying mouse model) towards cisplatin after treatment with combination of miR-125b and p53 containing plasmids.^25^

Interestingly, few miRNAs that were downregulated in expression in both p53/exo and combi/exo were associated with the pathway for ‘human T-lymphotrophic virus-1 infection'. The human T-lymphotrophic virus-1 belongs to a class of viruses called retroviruses and are associated with induction of cancer by retrotransposition of their DNA into host genome.^32^ Additionally, human T-lymphotrophic virus-1 is considered to be a risk factor for bronchioloalveolar carcinoma.^33^ A decrease in the miRNA associated with this pathway could also potentially indicate an overall epigenetic change against cancer development and progression mediated via altered miRNA profiles in the secreted exosome. It is noteworthy to mention that, although there are several other factors contained in the exosomes, we only focused on the miRNA profiles in the exosomes as mediators of cell-to-cell communications. Other secreted factors, for example cytokines, interleukins, growth factors, secreted in exosomes along with miRNA might contribute to the observed effects and can be identified in the future studies. However, the focus of the current study was to provide preliminary *in vitro* evidence for the novel therapeutic modality in form plasmid-encapsulated nanoparticles to induce apoptosis in the cancer cells and also reprogram the miRNA content in microvesicles, re-educating TME cells thus attaining enhanced antitumor efficacy.

## Conclusions

Exosomes have been considered as a novel delivery strategy, but their potential clinical translation has been hampered due to concerns with isolation and low yield, *ex vivo* manipulation and toxicity concerns with re-injection of modified exosomes in patients. In this study, we have investigated delivery of HA nanoparticles encapsulating plasmid DNA encoding wt-p53+miR-125b to evaluate their expression in the exosomes secreted by SK-LU-1 cancer cells. Along with modulation of wt-p53 and miR-125b expression these exosomes showed reprogramed global miRNA profile and activation of pathways associated with apoptosis as well as p53 signaling. Furthermore, altered miRNA profile also mediated macrophage repolarization towards a more pro-inflammatory/antitumor M1 phenotype. Further studies, especially *in vivo* studies, are warranted to assess the direct influence of tumor-associated macrophage repolarization on tumor growth suppression and the improvement in therapeutic effects. Exosome-mediated macrophage repolarization strategy could enable development of approaches that can affect both tumor cells and other cells in the tumor microenvironment by exploiting the ‘bystander effect'.

## Materials and methods

### Experimental design

The objective of the current study was to modify the exosomal content of SK-LU-1 lung cancer cells by transfecting SK-LU-1 cells with plasmid coding for wt-p53 as well as miR-125b. We also wanted to investigate if such modification in the secreted microvesicles, in specific in the miRNA profile, can induce macrophage reprogramming towards a more anti-inflammatory phenotype. We employed a non-viral drug-delivery system using HA-PEI/HA-PEG-based nanoparticles to encapsulate the different plasmids.

### Materials

HA with an average molecular weight of 20 kDa was obtained from Lifecore Biomedical Co. (Chaska, MN, USA). Poly(ethylene imine) (PEI MW 20 000 Da) was obtained from Polysciences Inc. (Warrington, PA, USA). Mono-functional poly(ethylene glycol)-amine (PEG_2K_-NH_2_, MW=2000 Da) was purchased from Creative PEG Works, Inc. (Winston Salem, NC, USA).

### microRNA-125b-2 expressing plasmid DNA preparation, isolation and purification

The microRNA construct for miRNASelect pEGP-mmu-mmu-miR-125b expression vector and Flag-Tag-wt-p53 as well as null-plasmid were obtained from (Cell Biolabs, Inc., CA, USA). The plasmids were provided as bacterial glycerol stock and stored at −80 °C. Twenty-five grams of the powdered LB Broth, Miller (Fisher BioReagents, Fair Lawn, NJ, USA) and 25 g of powdered media plus 15 g of Agar (Fisher Scientific, Pittsburg, PA, USA) was dissolved distilled water to make 1 l of LB media and LB agar media, respectively, followed by autoclave-based sterilization. Post-sterilization, the media was cooled to below 50 °C, and ampicillin was added (final concentration of 100 μg/ml). This prepared LB agar media was poured into 100 mm cell culture Petri dishes and allowed to settle at 4 °C. A sterile inoculation loop was used to streak the plasmid transformed in bacteria suspension onto an LB agar ampicillin plate. This was followed by incubating the plate at 37 °C for 16 h. Furthermore, five or six colonies were then picked into 15 ml of LB ampicillin media for a starter culture, followed by an overnight incubation at 37 °C for 16 h with shaking at 250 r.p.m. This was followed by dilution of starter culture at 1:500 ratio in LB ampicillin media and incubated at 37 °C for 16 h with shaking at 250 r.p.m. The QIAFilter Mega Kit (Qiagen, Valencia, Spain) was used for the purification and isolation of plasmid DNA. All plasmids were produced according to the manufacturer's protocol.

### Cell culture experiments

SK-LU-1 human lung adenocarcinoma cells were obtained from American Type Culture Collections (ATCC, Manassas, VA, USA). The cells were cultured in Dulbecco's modified Eagle's medium supplemented with 10% fetal bovine serum, 1% Pen–Strep and grown at 37 °C, 5% CO_2_. Similarly, J774 macrophage cell line was obtained from ATCC and grown in Dulbecco's modified Eagle's medium supplemented with 10% fetal bovine serum and 1% penicillin/streptomycin. Both the cell lines were maintained in an incubator at 37 °C and 5% CO_2_.

### Formulation and characterization of plasmid DNA-loaded HA nanoparticles

Combinatorial designed HA formulations were prepared according to the protocol previously published.^25, 34, 35^ Briefly, the HA-PEI and HA-PEG conjugates were prepared using this combinatorial approach. For the synthesis of HA-PEG, 50 mg of maleimide-PEG-amine was added to EDC/NHS activated HA. The HA-PEI and HA-PEG solutions (3 mg/ml) were prepared by dissolving the polymer in phosphate-buffered saline. Nanoparticle size and charge were determined on a Malvern Nano ZS (Malvern Instruments). Transmission electron microscopy (JEOL, JEM-1000, Tokyo, Japan) was performed to assess the formation of plasmid-loaded nanoparticles. Uranyl acetate ribonucleic acid stain was used to differentiate the plasmid DNA from the polymer. The ability of these nanoparticle complexes to release the plasmid by ion-exchange was determined by treating them with poly(acrylic acid), followed by gel electrophoresis. Encapsulation efficiency of plasmid in the nanoparticle was assessed using Picogreen fluorescence assay.

### Quantitative assessment of plasmid-encapsulated HA nanoparticle uptake

SK-LU-1 cells (0.2 million) were plated overnight in six-well plates and treated with 20 μg plasmid DNA-encapsulated HA-PEI/HA-PEG nanoparticles. Transgene expression was assessed following 24 and 48 h as previously described.^25^

### Exosome isolation

Exosomes were isolated using the total exosome isolation kit (Life Technologies, Carlsbad, CA, USA) according to the manufacturer's instructions. Briefly, cell supernatants of 2 × 10^6^ SK-LU-1 cells cultured in Dulbecco's modified Eagle's medium with 10% exosome-depleted fetal bovine serum (Systems Biosciences, Inc., Palo Alto, CA, USA) were collected and centrifuged at 2000 *g* for 30 min to remove cell debris. The supernatants were added to the total exosome isolation reagent (0.5 volume) and incubated overnight at 4 **°**C. After incubation the samples were centrifuged at 10 000 *g* for 1 h at 4 °C. The supernatants were aspirated and the exosome pellets were resuspended in sterile 1 × phosphate-buffered saline. We have designated the isolated the exosomes as SK/exo, 125b/exo, p53/exo and combi/exo based on isolation from untreated cells, cells transfected with plasmid DNA expressing miR-125b, cells transfected with plasmid DNA expressing wt-p53, and cells transfected with both plasmid DNA vectors, respectively.

### Exosome size and morphology assessment

The size and morphology of exosomes isolated from untreated and transfected cells were determined by transmission electron microscopy (JEOL). Briefly, a 20-μl aliquot of the suspension was loaded onto a carbon-coated grid for 2 min at room temperature. The grid was positioned with the coating side facing the drop containing exosomes. The samples were fixed by covering the grid with 10 μl of 1% aqueous phosphotungstic acid for 1 min and then observed under a transmission electron microscope (Hitachi, Shiga, Japan).

### Exosomal proteins and RNA isolation

Exosomal proteins and RNA isolation were extracted using the RIPA lysis buffer (Biouniquer Technology, Nanjing, China) and Total Exosome RNA and Protein Isolation Kit (Life Technologies) according to the manufacturer's instructions, respectively. The total RNA concentration was measured on a NanoDrop 2000 spectrophotometry (Thermo Scientific, Waltham, MA, USA).

### Protein quantification in isolated exosomes

The isolated and purified exosomes were resuspended in 1 × phosphate-buffered saline and quantified by their protein concentration using Micro BCA Protein Assay Kit (Pierce Biotechnology, Inc., Rockford, IL, USA). The protein concentrations of exosomes were determined by following the manufacturer protocol.

### TaqMan miRNA quantitative real-time PCR analysis

qPCR was performed using TaqMan miRNA assays (Life Technologies). cDNA for each miRNA of interest was synthesized from an input of 5 ng of total RNA using the TaqMan microRNA Reverse Transcription Reagents (Life Technologies) and specific reverse transcription primers (Life Technologies). Real-time PCR with TaqMan probes was performed on a Roche Lightcycler using the following conditions: 10 min at 95 °C, followed by 40 cycles of 95 °C for 30 s and 60 °C for 1 min. All assays were performed in triplicates. Triplicate CT values were averaged and normalized to the geometric mean of U6 mRNA levels, which was selected as endogenous controls based on previous preliminary experiments (data not shown). The normalized relative expression was calculated as Δ(ΔCT). CT values >36 were considered to be below the limit of detection.

### miRNA analysis in exosomes from cell medium

Exosomes were isolated as mentioned above. Small RNA was purified from exosomes using the mirVana isolation kit (Life Technologies). The small RNA concentration and quality were determined by BioAnalyzer 2100 (Agilent Technologies, Santa Clara, CA, USA), and at least 5 ng RNA were then used as input for the nCounter Human miRNA Expression Assay kit (NanoString Technologies, Seattle, WA, USA). The miRNA expression profiles were analyzed according to the manufacturer's instructions.

### NanoString nCounter System miRNA Assay

One hundred nanograms of total RNA from each sample was provided to the Microarray Centre for NanoString nCounter analysis. The samples were prepared for nCounter miRNA expression profiling according to the manufacturer's recommendations (NanoString). For each sample, a scan of 600 fields of view was imaged. Before data normalization, nCounter data imaging QC metrics were assessed. There was no significant discrepancy between the fields of view attempted and the fields of view counted. The binding density for the samples ranged between 0.24 and 0.72—within the typical recommended range. The raw data were loaded into the R statistical environment (v.2.14.0), and reannotated against miRBase v.16. First, probes indicated to have some level of background were corrected using probe level adjustment factors. Then, the geometric mean of the positive controls was used for code count normalization, while the background was estimated using the mean of the negative controls. Sample input amounts were normalized to the geometric mean of five housekeeping mRNA controls (ACTB, B2M, GAPDH, RPL19 and RPL10) included in the assay, and finally to total miRNA count. Atleast 30% of the total miRNAs for each array data set were detected in at least two out of three biological replicates of each group.

### Qualitative transfection efficiency using western blot

Protein expression for flag-p53 was evaluated using western blot as previously described.^36^ Briefly, proteins were extracted from tumors using a Total Protein Extraction Kit (Millipore, Billerica, MA, USA) and a Powergen 125 tissue homogenizer (Fisher Scientific, Waltham, MA, USA). Tissue lysate samples were analyzed for total protein concentration using the BCA assay (Pierce). Fifty micrograms of total protein extract was run on a precast 4–20% sodium dodecyl sulfate–polyacrylamide gel electrophoresis system at 200 V for 30 min. Subsequently, protein bands on the gel were transferred onto a PVDF membrane by an iBlot Dry Blotting System (Life Technologies). The membrane was blocked with 5% milk in Tween-containing Tris buffer saline (TBS-t) for 1 h at room temperature. Membrane was cut and incubated with 1:1000 dilution of primary rabbit β-actin antibody or 1:1000 dilution of primary mouse monoclonal antiFLAGM2 antibody (Sigma-Aldrich, St Louis, MO, USA) separately overnight at 4 °C. Membranes were then washed three times with TBS-t and incubated with 1:2000 dilutions of secondary anti-rabbit or anti-mouse horseradish peroxidase-conjugated IgG (Cell Signaling Technology Inc., Danvers, MA, USA) in TBS-t for 1 h at room temperature. After rinsing excess antibody with TBS-t and water, 4 ml ECL substrate (Pierce) was added and mixed with membranes for 5 min, which is cleaved by peroxidase to give a chemiluminescent product. The membranes were visualized using a Kodak Digital X-ray Specimen (DXS) System. β-Actin was used as a protein loading control. Quantification was performed using Image J software and ratios were calculated respective to the beta-actin concentrations.

### Repolarization of J774.A1 macrophages following incubation with isolated exosomes

SK-LU-1 cells were transfected with plasmid-encapsulated HA nanoparticles (miR-125b and p53) as single or combination therapy. Forty-eight hours post transfection the 80 μg of p53/exo, 125b/exo and combi/exo were isolated from the SK-LU-1 cells following treatment. The exosome-treated J774 cells were collected after 24 h and M1 and M2 gene expression was assessed by qRT–PCR. RNA was isolated and cDNA was synthesized as described previously.^37^

### Statistical data analysis

All data analyses and graphical representations were performed and generated in the GraphPad Prism 6.0. Agglomerative hierarchical clustering was performed using Spearman's correlation coefficients as input, Euclidean distance as the distance metric and complete linkage. Results were visualized with heatmaps using DIANA miRPATH V.2.0. The data were analyzed using the Mann–Whitney *U*-test and Steel test to analyze statistical differences. The *P*-values were adjusted for multiple testing using the false discovery rate approach. Significant miRNAs were selected based on an arbitrary |fold change|⩾2 and *P* adjusted⩽0.05.

## Figures and Tables

**Table 1:** MicroRNAs associated functional pathways in SK/exo

*#*	*KEGG pathway*	*# of genes*	*# of microRNAs*	P*-value*
1	Glycosaminoglycan biosynthesis (hsa00532)	154	31	<1e-16
2	TGF-beta signaling pathway (hsa04350)	78	29	4.33E-06
3	Focal adhesion (hsa04510)	124	25	3.96E-05
4	Wnt signaling pathway (hsa04310)	76	21	2.47E-04
5	Ubiquitin mediated proteolysis (hsa04120)	47	17	2.10E-04
6	Non-small cell lung cancer (hsa05223)	52	23	1.38E-04

The exosomes secreted from SK-LU-1 cells were collected and miRNA profiling was performed. Using microT-CDs target-predicting data set along with mirPATH software, we characterized the top KEGG pathways predicted for interaction enrichments for miRNAs in SK/exo. Ln(*P*-value) is a natural logarithmic scale of the *P*-value obtained from a *χ*^2^ test comparing expected number of genes with interaction and the actual number composing the pathways (DIANA algorithm).

**Table 2:** MicroRNA-associated functional pathways in p53/exo

*#*	*KEGG pathway*	*# of genes*	*# of microRNAs*	P*-value*
1	PI3K–Akt signaling pathway (hsa04151)	203	27	<1e-16
2	Focal adhesion (hsa04510)	124	25	<1e-16
3	Ubiquitin-mediated proteolysis (hsa04120)	91	24	<1e-16
4	Pathways in cancer (hsa05200)	192	24	<1e-16
5	MAPK signaling pathway (hsa04010)	141	23	<1e-16
6	ErbB signaling pathway (hsa04012)	59	22	<1e-16
7	TGF-beta signaling pathway (hsa04350)	60	21	<1e-16
8	p53 signaling pathway (hsa04115)	48	21	<1e-16
9	Wnt signaling pathway (hsa04310)	97	20	<1e-16
10	Small cell lung cancer (hsa05222)	49	20	<1e-16
11	Transcriptional misregulation in cancer (hsa05202)	90	18	<1e-16
12	Chronic myeloid leukemia (hsa05220)	43	17	5.52E-13
13	Phosphatidylinositol signaling system (hsa04070)	40	17	1.02E-09
14	HTLV-I infection (hsa05166)	121	17	1.48E-07
15	Endocytosis (hsa04144)	99	16	8.47E-09
16	Melanoma (hsa05218)	47	16	6.33E-13
17	Regulation of actin cytoskeleton (hsa04810)	100	13	7.73E-09
18	Non-small-cell lung cancer (hsa05223)	30	11	1.12E-08
19	HIF-1 signaling pathway (hsa04066)	52	10	0.002182597
20	ECM–receptor interaction (hsa04512)	28	7	<1e-16

We collected the exosomes secreted from SK-LU-1 cells transfected with HA nanoparticles encapsulated with wt-p53 plasmid. miRNA expression was undertaken using Nanostring. Using microT-CDs target predicting data set along with mirPATH software, we characterized the top KEGG pathways predicted for interaction enrichments for miRNAs in p53/exo. Ln(*P*-value) is a natural logarithmic scale of the *P*-value obtained from a *χ*^2^ test comparing expected number of genes with interaction and the actual number composing the pathways (DIANA algorithm). The third column indicates the number of genes identified as targets of the differentially expressed miRNAs in the specific groups enlisted in the fourth column. Abbreviations: ECM, extracellular matrix; HIF-1, hypoxia-inducible factor-1; HTLV-1, human T-lymphotrophic virus-1

.

**Table 3:** MicroRNA-associated functional pathways in 125b/exo

*#*	*KEGG pathway*	*# of genes*	*# of microRNAs*	P*-value*
1	Focal adhesion (hsa04510)	119	55	1.48E-17
2	PI3K–Akt signaling pathway (hsa04151)	189	54	2.60E-14
3	MAPK signaling pathway (hsa04010)	135	50	4.09E-11
4	mTOR signaling pathway (hsa04150)	40	47	1.05E-08
5	Ubiquitin mediated proteolysis (hsa04120)	80	44	4.76E-10
6	p53 signaling pathway (hsa04115)	43	40	2.10E-08
	Calcium signaling pathway (hsa04020)	48	40	2.30E-03
7	ErbB signaling pathway (hsa04012)	55	39	3.43E-10
8	HTLV-1 infection (hsa05166)	84	38	5.09E-05
9	Pathways in cancer (hsa05200)	172	36	2.91E-08
10	Wnt signaling pathway(hsa04310)	92	36	5.17E-07
11	Melanoma (hsa05218)	44	36	1.54E-07
12	Transcriptional misregulation in cancer (hsa05202)	93	34	1.22E-05
13	Colorectal cancer (hsa05210)	37	34	4.21E-05
14	Small cell lung cancer (hsa05222)	49	32	2.74E-13
15	TGF-beta signaling pathway (hsa04350)	57	31	2.65E-12
16	Renal cell carcinoma (hsa05211)	43	30	4.07E-09
17	ECM–receptor interaction (hsa04512)	35	26	4.82E-113
18	Glycosaminoglycan biosynthesis—chondroitin sulfate (hsa00532)	9	15	4.43E-10

We collected the exosomes secreted from SK-LU-1 cells transfected with HA nanoparticles encapsulated with miR-125b plasmid. miRNA expression was undertaken using Nanostring. Using microT-CDs target predicting data set along with mirPATH software, we characterized the top KEGG pathways predicted for interaction enrichments for miRNAs in 125b/exo. Ln(*P*-value) is a natural logarithmic scale of the *P*-value obtained from a *χ*^2^ test comparing expected number of genes with interaction and the actual number composing the pathways (DIANA algorithm). The third column indicates the number of genes identified as targets of the differentially expressed miRNAs in the specific groups enlisted in the fourth column. Abbreviations: ECM, extracellular matrix; HTLV-1, human T-lymphotrophic virus-1.

**Table 4:** miRNA-associated functional pathways in combi/exo

*#*	*KEGG pathway*	*# of genes*	*# of microRNAs*	P*-value*
1	ECM–receptor interaction (hsa04512)	38	18	4.82E-113
2	Pathways in cancer (hsa05200)	207	34	1.35E-17
3	Focal adhesion (hsa04510)	128	34	1.48E-17
4	Transcriptional misregulation in cancer (hsa05202)	109	27	4.47E-16
5	Ubiquitin mediated proteolysis (hsa04120)	90	32	1.98E-14
6	PI3K–Akt signaling pathway (hsa04151)	208	35	2.60E-14
7	Small cell lung cancer (hsa05222)	50	23	2.74E-13
8	Endocytosis (hsa04144)	117	22	3.80E-13
9	ErbB signaling pathway (hsa04012)	63	34	5.51E-12
10	MAPK signaling pathway (hsa04010)	164	37	4.09E-11
11	p53 signaling pathway (hsa04115)	49	32	2.72E-09
12	Regulation of actin cytoskeleton (hsa04810)	124	28	1.83E-08
13	Cytokine–cytokine receptor interaction (hsa04060)	54	12	6.12E-07
14	Fc gamma R-mediated phagocytosis (hsa04666)	52	18	7.97E-07
15	NF-kappa B signaling pathway (hsa04064)	12	4	1.14E-06
16	T-cell receptor signaling pathway (hsa04660)	50	12	2.52E-06
17	HIF-1 signaling pathway (hsa04066)	59	15	1.75E-05
18	HTLV-I infection (hsa05166)	135	30	0.000172889
19	Notch signaling pathway (hsa04330)	30	12	0.000292382
20	Toll-like receptor signaling pathway (hsa04620)	23	5	0.000409522
21	VEGF signaling pathway (hsa04370)	30	10	0.000431036
22	Chemokine signaling pathway (hsa04062)	72	10	0.000437235
23	Cell adhesion molecules (CAMs) (hsa04514)	34	8	0.000764716
24	Natural killer cell mediated cytotoxicity (hsa04650)	21	3	0.001346897
25	Apoptosis (hsa04210)	32	7	0.008855041

We collected the exosomes secreted from SK-LU-1 cells transfected with HA nanoparticles encapsulated with wt-p53 plasmid in combination with HA nanoparticles encapsulating miR-125b plasmid. miRNA expression was undertaken using Nanostring. Using microT-CDs target predicting data set along with mirPATH software, we characterized the top KEGG pathways predicted for interaction enrichments for miRNAs in combi/exo. Ln(*P*-value) is a natural logarithmic scale of the *P*-value obtained from a *χ*^2^ test comparing expected number of genes with interaction and the actual number composing the pathways (DIANA algorithm). The third column indicates the number of genes identified as targets of the differentially expressed miRNAs in the specific groups enlisted in the fourth column.Abbreviations: ECM, extracellular matrix; HIF-1, hypoxia-inducible factor-1; HTLV-1, human T-lymphotrophic virus-1; VEGF, vascular endothelial growth factor.
