# Supplementary figures and images for: Modification of tumor cell exosome content by transfection with wt-p53 and microRNA-125b expressing plasmid DNA and its effect on macrophage polarization
Source: Oncogenesis. 2016 Aug 8;5(8):e250–. doi: 10.1038/oncsis.2016.52 (PMC5007827; doi:10.1038/oncsis.2016.52)

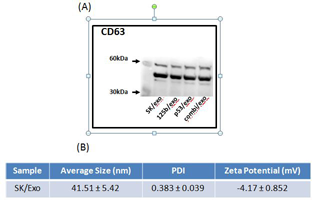

Supplement: Supplementary Figure 1 [file oncsis201652x1.tif]

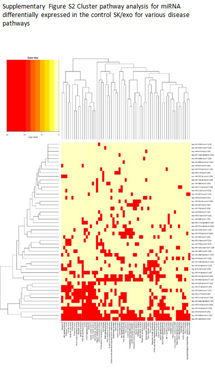

Supplement: Supplementary Figure 2 [file oncsis201652x2.tif]

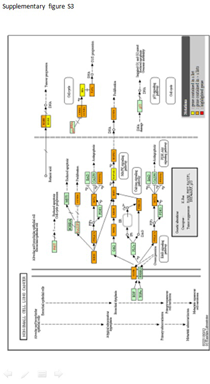

Supplement: Supplementary Figure 3 [file oncsis201652x3.tif]

## Slide 1
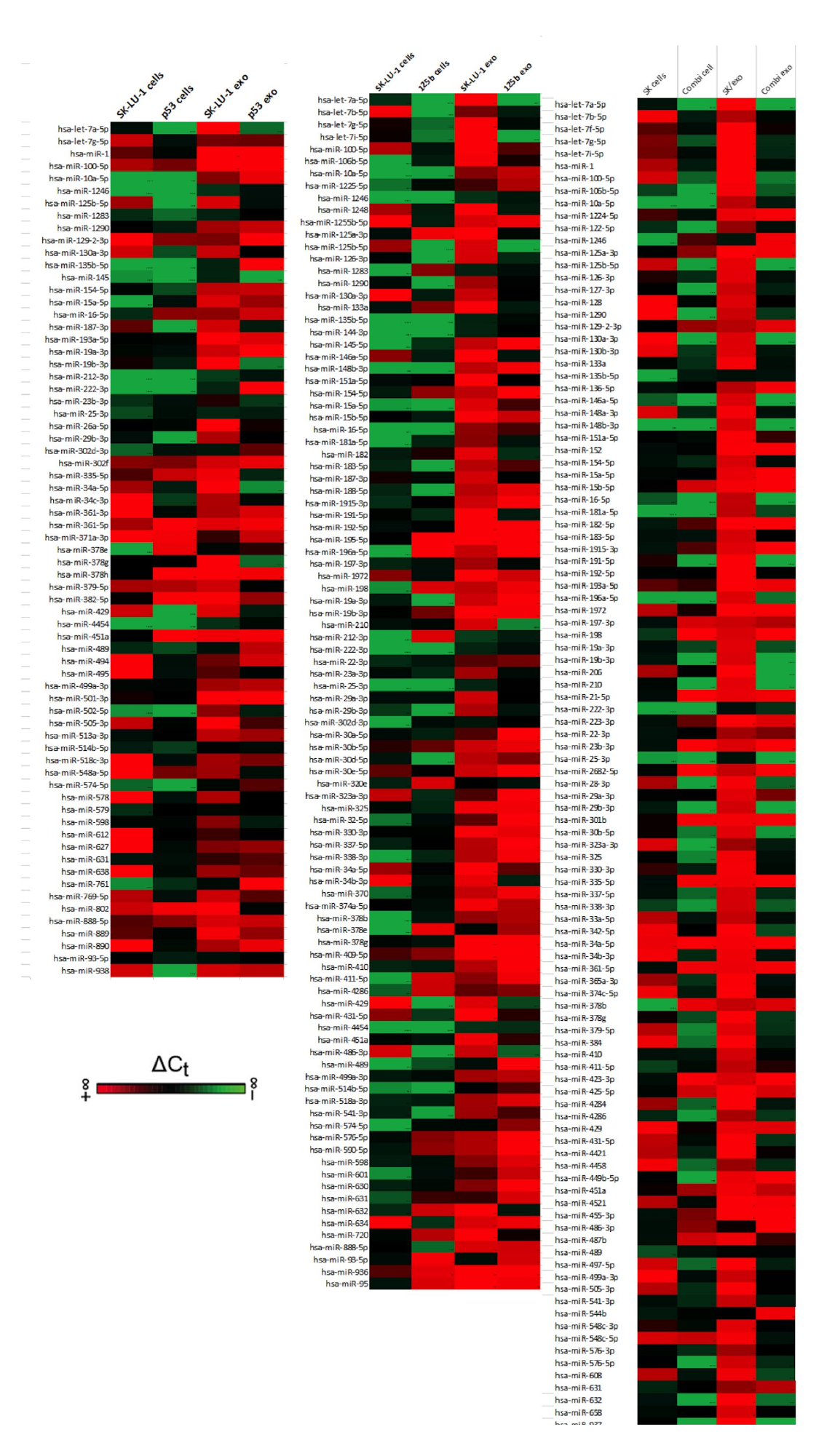

Supplement: Supplementary Figure 4 [file oncsis201652x4.ppt]

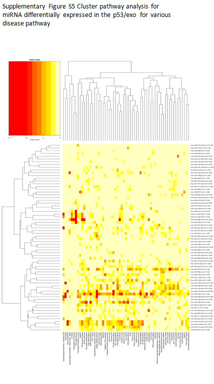

Supplement: Supplementary Figure 5 [file oncsis201652x5.tif]

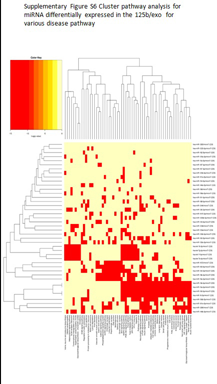

Supplement: Supplementary Figure 6 [file oncsis201652x6.tif]

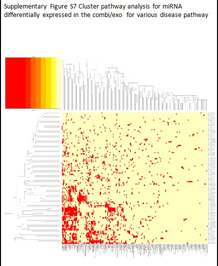

Supplement: Supplementary Figure 7 [file oncsis201652x7.tif]

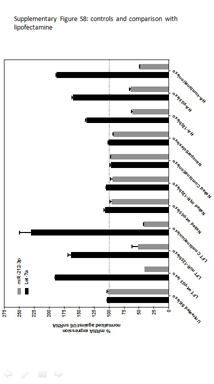

Supplement: Supplementary Figure 8 [file oncsis201652x8.tif]

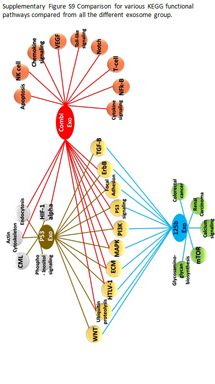

Supplement: Supplementary Figure 9 [file oncsis201652x9.tif]
