# Supplementary material for: Modification of tumor cell exosome content by transfection with wt-p53 and microRNA-125b expressing plasmid DNA and its effect on macrophage polarization
Source: Oncogenesis. 2016 Aug 8;5(8):e250–. doi: 10.1038/oncsis.2016.52 (PMC5007827; doi:10.1038/oncsis.2016.52)
Supplement: Supplementary Figure Legends [file oncsis201652x10.docx]

**SUPPLEMENTARY FIGURE LEGENDS**

**Figure S1.** Characterization of isolated exosomes. **(A)** Western blot identification by SDS-PAGE of protein found in vesicles. Proteins were separated on a 4–20% gradient SDS-PAGE gel under reducing conditions. The gel was Western blotted onto nitrocellulose membranes and probed with antibodies against the CD63 protein generally used a marker for microvesicles. **(B)** Malvern data for the size measurements of the exosomes isolated using a Malvern Zeta-sizer. The average particle size was observed to be 41.51 ± 3.42 nm.

**Figure S2.** Hierarchical cluster pathway analysis for microRNA differentially expressed in the control SK-LU-1 exosomes (SK/exo) for various disease pathways. Ln(*p*-value) is a natural logarithmic scale of the *p*-value obtained from a χ-square test comparing expected number of genes with interaction and the actual number composing the pathways (DIANA algorithm).

**Figure S3.** Specific genes identified in the non-small cell lung cancer signaling pathway as targets of microRNAs characterized from SK-LU-1 exosomes (SK/exo). The data indicates that exosomes in SK-LU-1 have microRNA compositions that can propogate cancer growth and metastasis. Functional pathway diagram obtained from KEGG GO image using DIANA Algorith Tools.

**Figure S4.** microRNA analysis in SK-LU-1 cells and their exosomes pre- and post-treatment with HA-nanoparticles containing p53 and / or miR-125b plasmids. **(A)** Selected miRNAs (indicated as miRs) increased or decreased in p53/cell and p53/exo **(B)** 125b/cell and 125b/exo **(C)** combi/cell and combi/exo, analyzed by Nanostring analysis. Mean-centered heat-map showing unsupervised hierarchical clustering of miRNA levels in UT SKLU-1 cells and SK/exo and the respective treatments. P53/cells and P53/exo – CD44 targeting nanoparticles encapsulating p53 plasmid treated SK-LU-1 cells and exosomes collected thereof; 125b/cells and 125b/exo– CD44 targeting nanoparticles encapsulating 125b plasmid treated SK-LU-1 cells and exosomes collected thereof, combi/cells and combi/exo CD44 targeted nanoparticles encapsulating p53 or miR-125b encoding plasmid given as combination therapy with an 18 hours of time lag.

**Figure S5.** Hierarchical cluster pathway analysis for microRNA differentially expressed in the p53/exo for various KEGG functional pathways. Ln(*p*-value) is a natural logarithmic scale of the *p*-value obtained from a χ-square test comparing expected number of genes with interaction and the actual number composing the pathways (DIANA algorithm).

**Figure S6.** Hierarchical cluster pathway analysis for miRNA differentially expressed in the 125b/exo for various KEGG functional pathways. Ln(*p*-value) is a natural logarithmic scale of the *p*-value obtained from a χ-square test comparing expected number of genes with interaction and the actual number composing the pathways (DIANA algorithm).

**Figure S7.** Hierarchical cluster pathway analysis for miRNA differentially expressed in the combi/exo for various KEGG functional pathways. Ln(*p*-value) is a natural logarithmic scale of the *p*-value obtained from a χ-square test comparing expected number of genes with interaction and the actual number composing the pathways (DIANA algorithm).

**Figure S8.** To compare the transfection efficiency of HA-nanoparticles with Lipofectamine transfection reagent as well as internal naked plasmid controls as well as empty nanoparticles. SK-LU-1 cells were treated with different treatment regimens and exosomes were collected. microRNA levels of miR-212-3p and let-7a were evaluated in these exosomes using Taqman® probes after different treatment regimes. n=6, Mean +/- SEM

**Figure S9.** Comparison for various KEGG functional pathways compared from all the different exosome group. The different KEGG functional pathway groups were aligned manually for understanding the functional relationship between different exosomes group. The pathways shared by all the groups were aligned in the center and the pathways mutually exclusive were spaced towards the periphery.
