# Supplementary material for: Modification of tumor cell exosome content by transfection with wt-p53 and microRNA-125b expressing plasmid DNA and its effect on macrophage polarization
Source: Oncogenesis. 2016 Aug 8;5(8):e250–. doi: 10.1038/oncsis.2016.52 (PMC5007827; doi:10.1038/oncsis.2016.52)
Supplement: Supplementary Table 1 [file oncsis201652x11.docx]

| Supplementary Table S1  List of Differentially expressed microRNAs in SK/exosomes | | | |
| --- | --- | --- | --- |
| **MicroRNAs** | **Nanostring code quantities** |  |  |
| hsa-miR-212-3p | 2048 |  |  |
| hsa-miR-1246 | 1731 |  |  |
| hsa-miR-4454 | 1699 |  |  |
| hsa-miR-25-3p | 1366 |  |  |
| hsa-miR-144-3p | 1298 |  |  |
| hsa-miR-1283 | 1224 |  |  |
| hsa-miR-302d-3p | 836 |  |  |
| hsa-miR-489 | 634 |  |  |
| hsa-miR-579 | 354 |  |  |
| hsa-miR-574-5p | 353.6 |  |  |
| hsa-miR-378e | 342 |  |  |
| hsa-miR-514b-5p | 309 |  |  |
| hsa-miR-320e | 231.8 |  |  |
| hsa-miR-222-3p | 227.8 |  |  |
| hsa-miR-720 | 216 |  |  |
| hsa-miR-297 | 174.34 |  |  |
| hsa-miR-631 | 170.55 |  |  |
| hsa-miR-371a-3p | 170.19 |  |  |
| hsa-miR-612 | 163.2 |  |  |
| hsa-miR-1225-5p | 162.97 |  |  |
| hsa-miR-217 | 161 |  |  |
| hsa-miR-495 | 148.2 |  |  |
| hsa-miR-22-3p | 147.2 |  |  |
| hsa-let-7g-5p | 143.5 |  |  |
| hsa-miR-32-5p | 142.6 |  |  |
| hsa-miR-4286 | 140.4 |  |  |
| hsa-miR-548aa | 138 |  |  |
| hsa-miR-494 | 133.4 |  |  |
| hsa-miR-135b-5p | 132.65 |  |  |
| hsa-miR-215 | 132.6 |  |  |
| hsa-miR-570-3p | 129.2 |  |  |
| hsa-miR-216a | 128.86 |  |  |
| hsa-miR-129-2-3p | 125.07 |  |  |
| hsa-miR-487a | 124.2 |  |  |
| hsa-miR-627 | 121.28 |  |  |
| hsa-miR-544a | 119.6 |  |  |
| hsa-miR-10a-5p | 118.9 |  |  |
| hsa-miR-598 | 117.49 |  |  |
| hsa-miR-3182 | 115.6 |  |  |
| hsa-miR-411-5p | 115.2 |  |  |
| hsa-miR-369-5p | 115 |  |  |
| hsa-miR-502-5p | 113.7 |  |  |
| hsa-miR-16-5p | 112.8 |  |  |
| hsa-miR-450b-5p | 110.2 |  |  |
| hsa-miR-515-5p | 110.2 |  |  |
| hsa-miR-33a-5p | 109.91 |  |  |
| hsa-miR-378b | 109.91 |  |  |
| hsa-miR-2682-5p | 108.3 |  |  |
| hsa-miR-1287 | 105.8 |  |  |
| hsa-miR-638 | 105.8 |  |  |
| hsa-miR-548ai | 105.4 |  |  |
| hsa-miR-548a-5p | 104.5 |  |  |
| hsa-miR-23a-3p | 102.6 |  |  |
| hsa-miR-512-3p | 102.6 |  |  |
| hsa-miR-197-3p | 102 |  |  |
| hsa-miR-1290 | 101.2 |  |  |
| hsa-miR-499a-3p | 101.2 |  |  |
| hsa-miR-518c-3p | 101.2 |  |  |
| hsa-miR-767-3p | 101.2 |  |  |
| hsa-miR-890 | 98.54 |  |  |
| hsa-miR-1255b-5p | 96.6 |  |  |
| hsa-miR-376c | 96.6 |  |  |
| hsa-miR-361-3p | 96.2 |  |  |
| hsa-miR-302e | 95.2 |  |  |
| hsa-miR-34c-3p | 95.2 |  |  |
| hsa-miR-4516 | 95.2 |  |  |
| hsa-miR-578 | 95.2 |  |  |
| hsa-miR-3190-5p | 95 |  |  |
| hsa-miR-590-5p | 95 |  |  |
| hsa-miR-29b-3p | 94.75 |  |  |
| hsa-miR-1272 | 92 |  |  |
| hsa-miR-338-5p | 92 |  |  |
| hsa-miR-595 | 92 |  |  |
| hsa-miR-410 | 91.64 |  |  |
| hsa-miR-337-5p | 90.96 |  |  |
| hsa-miR-548ad | 90.96 |  |  |
| hsa-miR-188-5p | 90 |  |  |
| hsa-miR-148b-3p | 87.5 |  |  |
| hsa-miR-1234 | 87.4 |  |  |
| hsa-miR-198 | 87.4 |  |  |
| hsa-miR-3187-3p | 87.4 |  |  |
| hsa-miR-423-3p | 87.4 |  |  |
| hsa-miR-499a-5p | 87.4 |  |  |
| hsa-miR-520e | 87.4 |  |  |
| hsa-miR-562 | 87.4 |  |  |
| hsa-miR-761 | 87.4 |  |  |
| hsa-miR-942 | 87.4 |  |  |
| hsa-miR-154-5p | 87.17 |  |  |
| hsa-miR-513a-3p | 87.17 |  |  |
| hsa-miR-145-5p | 85 |  |  |
| hsa-miR-485-3p | 85 |  |  |
| hsa-miR-199a-5p | 83.38 |  |  |
| hsa-miR-1205 | 82.8 |  |  |
| hsa-miR-196a-5p | 82.8 |  |  |
| hsa-miR-23b-3p | 82.8 |  |  |
| hsa-miR-504 | 82.8 |  |  |
| hsa-miR-581 | 82.8 |  |  |
| hsa-miR-370 | 82.36 |  |  |
| hsa-miR-130a-3p | 81.6 |  |  |
| hsa-miR-323b-5p | 80.6 |  |  |
| hsa-miR-1200 | 79.8 |  |  |
| hsa-miR-548ah-5p | 79.8 |  |  |
| hsa-miR-548am-3p | 79.8 |  |  |
| hsa-miR-557 | 79.8 |  |  |
| hsa-miR-130b-3p | 79.59 |  |  |
| hsa-miR-30e-5p | 79.59 |  |  |
| hsa-miR-769-5p | 79.59 |  |  |
| hsa-miR-9-5p | 79.59 |  |  |
| hsa-miR-367-3p | 79.2 |  |  |
| hsa-miR-29a-3p | 78.2 |  |  |
| hsa-miR-550b-3p | 78.2 |  |  |
| hsa-miR-125a-5p | 78.2 |  |  |
| hsa-miR-19a-3p | 78.2 |  |  |
| hsa-miR-30b-5p | 78.2 |  |  |
| hsa-miR-379-5p | 78.2 |  |  |
| hsa-miR-548c-3p | 78.2 |  |  |
| hsa-miR-576-5p | 78.2 |  |  |
| hsa-miR-1288 | 76 |  |  |
| hsa-miR-3168 | 76 |  |  |
| hsa-miR-519c-3p | 76 |  |  |
| hsa-miR-876-5p | 76 |  |  |
| hsa-miR-938 | 76 |  |  |
| hsa-miR-1324 | 75.8 |  |  |
| hsa-miR-1827 | 75.8 |  |  |
| hsa-miR-187-3p | 75.8 |  |  |
| hsa-miR-27b-3p | 75.8 |  |  |
| hsa-miR-302f | 75.8 |  |  |
| hsa-miR-149-5p | 75 |  |  |
| hsa-miR-126-3p | 74.8 |  |  |
| hsa-miR-548d-3p | 74.8 |  |  |
| hsa-miR-137 | 73.6 |  |  |
| hsa-miR-2113 | 73.6 |  |  |
| hsa-miR-515-3p | 73.6 |  |  |
| hsa-miR-568 | 73.6 |  |  |
| hsa-miR-125b-5p | 72.2 |  |  |
| hsa-miR-634 | 72.2 |  |  |
| hsa-miR-888-5p | 72.2 |  |  |
| hsa-miR-127-5p | 71.4 |  |  |
| hsa-miR-378f | 71.4 |  |  |
| hsa-miR-519d | 71.4 |  |  |
| hsa-miR-15a-5p | 70.2 |  |  |
| hsa-miR-10b-5p | 69 |  |  |
| hsa-miR-147b | 69 |  |  |
| hsa-miR-548p | 69 |  |  |
| hsa-miR-1183 | 68.4 |  |  |
| hsa-miR-1224-5p | 68.4 |  |  |
| hsa-miR-196b-5p | 68.4 |  |  |
| hsa-miR-361-5p | 68.4 |  |  |
| hsa-miR-1908 | 68.22 |  |  |
| hsa-miR-345-5p | 68.22 |  |  |
| hsa-miR-492 | 68.22 |  |  |
| hsa-miR-520h | 68.22 |  |  |
| hsa-miR-548aj-3p | 68.22 |  |  |
| hsa-miR-542-3p | 68 |  |  |
| hsa-miR-934 | 68 |  |  |
| hsa-miR-1251 | 67.6 |  |  |
| hsa-miR-1323 | 67.2 |  |  |
| hsa-miR-141-3p | 67.2 |  |  |
| hsa-miR-1267 | 64.6 |  |  |
| hsa-miR-190b | 64.6 |  |  |
| hsa-miR-34b-3p | 64.6 |  |  |
| hsa-miR-487b | 64.6 |  |  |
| hsa-miR-762 | 64.6 |  |  |
| hsa-miR-892a | 64.6 |  |  |
| hsa-miR-1185-5p | 64.43 |  |  |
| hsa-miR-1245b-3p | 64.43 |  |  |
| hsa-miR-4455 | 64.43 |  |  |
| hsa-miR-485-5p | 64.43 |  |  |
| hsa-miR-1197 | 64.4 |  |  |
| hsa-miR-133b | 64.4 |  |  |
| hsa-miR-181c-5p | 64.4 |  |  |
| hsa-miR-185-5p | 64.4 |  |  |
| hsa-miR-450b-3p | 64.4 |  |  |
| hsa-miR-376a-3p | 63.69 |  |  |
| hsa-miR-1257 | 61.2 |  |  |
| hsa-miR-1825 | 61.2 |  |  |
| hsa-miR-3175 | 61.2 |  |  |
| hsa-miR-516a-3p | 61.2 |  |  |
| hsa-miR-548an | 61.2 |  |  |
| hsa-miR-587 | 61.2 |  |  |
| hsa-miR-630 | 61.2 |  |  |
| hsa-miR-1206 | 60.8 |  |  |
| hsa-miR-335-5p | 60.8 |  |  |
| hsa-miR-369-3p | 60.8 |  |  |
| hsa-miR-577 | 60.8 |  |  |
| hsa-miR-601 | 60.8 |  |  |
| hsa-miR-611 | 60.8 |  |  |
| hsa-miR-7-5p | 60.8 |  |  |
| hsa-miR-323a-5p | 60.64 |  |  |
| hsa-miR-325 | 60.64 |  |  |
| hsa-miR-451a | 60.64 |  |  |
| hsa-miR-548a-3p | 60.64 |  |  |
| hsa-miR-511 | 59.8 |  |  |
| hsa-miR-520a-5p | 59.8 |  |  |
| hsa-miR-98 | 59.8 |  |  |
| hsa-miR-105-5p | 58 |  |  |
| hsa-miR-142-3p | 58 |  |  |
| hsa-miR-1179 | 57.8 |  |  |
| hsa-miR-301b | 57.8 |  |  |
| hsa-miR-337-3p | 57.8 |  |  |
| hsa-miR-524-3p | 57.8 |  |  |
| hsa-miR-889 | 57.8 |  |  |
| hsa-miR-106b-5p | 57.6 |  |  |
| hsa-miR-15b-5p | 57 |  |  |
| hsa-miR-26b-5p | 57 |  |  |
| hsa-miR-28-3p | 57 |  |  |
| hsa-miR-299-5p | 57 |  |  |
| hsa-miR-520f | 57 |  |  |
| hsa-miR-548c-5p | 57 |  |  |
| hsa-miR-550a-5p | 57 |  |  |
| hsa-miR-662 | 57 |  |  |
| hsa-miR-767-5p | 57 |  |  |
| hsa-miR-95 | 55.49 |  |  |
| hsa-miR-1278 | 55.2 |  |  |
| hsa-miR-1306-3p | 55.2 |  |  |
| hsa-miR-1321 | 55.2 |  |  |
| hsa-miR-26a-5p | 55.2 |  |  |
| hsa-miR-328 | 55.2 |  |  |
| hsa-miR-34a-5p | 55.2 |  |  |
| hsa-miR-371b-3p | 55.2 |  |  |
| hsa-miR-548al | 55.2 |  |  |
| hsa-miR-624-3p | 55.2 |  |  |
| hsa-miR-195-5p | 55.1 |  |  |
| hsa-miR-503 | 55.1 |  |  |
| hsa-miR-1248 | 54.4 |  |  |
| hsa-miR-1284 | 54.4 |  |  |
| hsa-miR-323a-3p | 54.4 |  |  |
| hsa-miR-324-5p | 54.4 |  |  |
| hsa-miR-635 | 54.4 |  |  |
| hsa-let-7e-5p | 54 |  |  |
| hsa-miR-142-5p | 54 |  |  |
| hsa-miR-151a-5p | 53.2 |  |  |
| hsa-miR-1538 | 53.2 |  |  |
| hsa-miR-301a-3p | 53.2 |  |  |
| hsa-miR-338-3p | 53.2 |  |  |
| hsa-miR-339-3p | 53.2 |  |  |
| hsa-miR-365a-3p | 53.2 |  |  |
| hsa-miR-505-3p | 53.2 |  |  |
| hsa-miR-509-3-5p | 53.2 |  |  |
| hsa-miR-532-5p | 53.2 |  |  |
| hsa-miR-569 | 53.2 |  |  |
| hsa-miR-586 | 53.2 |  |  |
| hsa-miR-629-5p | 53.2 |  |  |
| hsa-miR-653 | 53.2 |  |  |
| hsa-miR-668 | 53.2 |  |  |
| hsa-miR-200b-3p | 53.06 |  |  |
| hsa-miR-3136-5p | 53.06 |  |  |
| hsa-miR-378g | 53.06 |  |  |
| hsa-miR-645 | 53.06 |  |  |
| hsa-miR-659-3p | 53.06 |  |  |
| hsa-let-7c | 52 |  |  |
| hsa-miR-378h | 51.3 |  |  |
| hsa-miR-27a-3p | 51 |  |  |
| hsa-miR-591 | 51 |  |  |
| hsa-miR-654-3p | 51 |  |  |
| hsa-miR-758 | 51 |  |  |
| hsa-miR-107 | 50.6 |  |  |
| hsa-miR-1266 | 50.6 |  |  |
| hsa-miR-1537 | 50.6 |  |  |
| hsa-miR-300 | 50.6 |  |  |
| hsa-miR-3123 | 50.6 |  |  |
| hsa-miR-3180-3p | 50.6 |  |  |
| hsa-miR-4461 | 50.6 |  |  |
| hsa-miR-449a | 50.6 |  |  |
| hsa-miR-610 | 50.6 |  |  |
| hsa-miR-656 | 50.6 |  |  |
| hsa-miR-802 | 50.6 |  |  |
| hsa-miR-1244 | 49.4 |  |  |
| hsa-miR-203 | 49.4 |  |  |
| hsa-miR-216b | 49.4 |  |  |
| hsa-miR-302c-3p | 49.4 |  |  |
| hsa-miR-329 | 49.4 |  |  |
| hsa-miR-409-5p | 49.4 |  |  |
| hsa-miR-4284 | 49.4 |  |  |
| hsa-miR-501-3p | 49.4 |  |  |
| hsa-miR-922 | 49.4 |  |  |
| hsa-miR-101-3p | 49.27 |  |  |
| hsa-miR-1258 | 49.27 |  |  |
| hsa-miR-1273d | 49.27 |  |  |
| hsa-miR-223-3p | 49.27 |  |  |
| hsa-miR-3178 | 49.27 |  |  |
| hsa-miR-421 | 49.27 |  |  |
| hsa-miR-432-5p | 49.27 |  |  |
| hsa-miR-4485 | 49.27 |  |  |
| hsa-miR-518a-3p | 49.27 |  |  |
| hsa-miR-548d-5p | 49.27 |  |  |
| hsa-miR-636 | 49.27 |  |  |
| hsa-miR-759 | 49.27 |  |  |
| hsa-let-7a-5p | 48 |  |  |
| hsa-let-7d-5p | 48 |  |  |
| hsa-let-7f-5p | 48 |  |  |
| hsa-miR-128 | 47.6 |  |  |
| hsa-miR-18b-5p | 47.6 |  |  |
| hsa-miR-372 | 47.6 |  |  |
| hsa-miR-375 | 47.6 |  |  |
| hsa-miR-4431 | 47.6 |  |  |
| hsa-miR-512-5p | 47.6 |  |  |
| hsa-miR-520c-3p | 47.6 |  |  |
| hsa-miR-545-3p | 47.6 |  |  |
| hsa-miR-548h-5p | 47.6 |  |  |
| hsa-miR-644a | 47.6 |  |  |
| hsa-miR-877-5p | 47.6 |  |  |
| hsa-miR-892b | 47.6 |  |  |
| hsa-miR-100-5p | 46.6 |  |  |
| hsa-miR-1243 | 46 |  |  |
| hsa-miR-146b-3p | 46 |  |  |
| hsa-miR-4521 | 46 |  |  |
| hsa-miR-552 | 46 |  |  |
| hsa-miR-590-3p | 46 |  |  |
| hsa-miR-615-3p | 46 |  |  |
| hsa-miR-887 | 46 |  |  |
| hsa-miR-891b | 46 |  |  |
| hsa-miR-93-5p | 46 |  |  |
| hsa-miR-937 | 46 |  |  |
| hsa-miR-1 | 45.9 |  |  |
| hsa-miR-1236 | 45.6 |  |  |
| hsa-miR-132-3p | 45.6 |  |  |
| hsa-miR-138-5p | 45.6 |  |  |
| hsa-miR-2054 | 45.6 |  |  |
| hsa-miR-2277-3p | 45.6 |  |  |
| hsa-miR-296-3p | 45.6 |  |  |
| hsa-miR-3200-3p | 45.6 |  |  |
| hsa-miR-342-3p | 45.6 |  |  |
| hsa-miR-563 | 45.6 |  |  |
| hsa-miR-625-5p | 45.6 |  |  |
| hsa-miR-1180 | 45.48 |  |  |
| hsa-miR-1264 | 45.48 |  |  |
| hsa-miR-139-5p | 45.48 |  |  |
| hsa-miR-219-2-3p | 45.48 |  |  |
| hsa-miR-497-5p | 45.48 |  |  |
| hsa-miR-524-5p | 45.48 |  |  |
| hsa-miR-548t-5p | 45.48 |  |  |
| hsa-miR-571 | 45.48 |  |  |
| hsa-miR-641 | 45.48 |  |  |
| hsa-miR-1297 | 44.2 |  |  |
| hsa-miR-1303 | 44.2 |  |  |
| hsa-miR-1470 | 44.2 |  |  |
| hsa-miR-206 | 44.2 |  |  |
| hsa-miR-3192 | 44.2 |  |  |
| hsa-miR-412 | 44.2 |  |  |
| hsa-miR-431-5p | 44.2 |  |  |
| hsa-miR-554 | 44.2 |  |  |
| hsa-miR-125a-3p | 43.7 |  |  |
| hsa-miR-770-5p | 43.7 |  |  |
| hsa-miR-1262 | 41.8 |  |  |
| hsa-miR-127-3p | 41.8 |  |  |
| hsa-miR-1273g-5p | 41.8 |  |  |
| hsa-miR-1322 | 41.8 |  |  |
| hsa-miR-193a-5p | 41.8 |  |  |
| hsa-miR-214-3p | 41.8 |  |  |
| hsa-miR-221-3p | 41.8 |  |  |
| hsa-miR-374c-5p | 41.8 |  |  |
| hsa-miR-423-5p | 41.8 |  |  |
| hsa-miR-4531 | 41.8 |  |  |
| hsa-miR-484 | 41.8 |  |  |
| hsa-miR-541-3p | 41.8 |  |  |
| hsa-miR-548l | 41.8 |  |  |
| hsa-miR-553 | 41.8 |  |  |
| hsa-miR-936 | 41.8 |  |  |
| hsa-miR-1260a | 41.69 |  |  |
| hsa-miR-1268b | 41.69 |  |  |
| hsa-miR-152 | 41.69 |  |  |
| hsa-miR-181a-5p | 41.69 |  |  |
| hsa-miR-194-5p | 41.69 |  |  |
| hsa-miR-2116-5p | 41.69 |  |  |
| hsa-miR-330-5p | 41.69 |  |  |
| hsa-miR-409-3p | 41.69 |  |  |
| hsa-miR-507 | 41.69 |  |  |
| hsa-miR-566 | 41.69 |  |  |
| hsa-miR-663b | 41.69 |  |  |
