# Supplementary material for: Modification of tumor cell exosome content by transfection with wt-p53 and microRNA-125b expressing plasmid DNA and its effect on macrophage polarization
Source: Oncogenesis. 2016 Aug 8;5(8):e250–. doi: 10.1038/oncsis.2016.52 (PMC5007827; doi:10.1038/oncsis.2016.52)
Supplement: Supplementary Table 2 [file oncsis201652x12.docx]

| p53/ exo decreased | Nanostring counts |
| --- | --- |
| hsa-miR-1 | 37 |
| hsa-miR-222-3p | 45 |
| hsa-miR-761 | 48 |
| hsa-miR-135b-5p | 49 |
| hsa-miR-129-2-3p | 53 |
| hsa-miR-19a-3p | 55 |
| hsa-miR-10a-5p | 63 |
| hsa-miR-890 | 63 |
| hsa-miR-302f | 64 |
| hsa-miR-361-3p | 72 |
| hsa-miR-494 | 75 |
| hsa-miR-16-5p | 79 |
| hsa-miR-154-5p | 82 |
| hsa-miR-1290 | 83 |
| hsa-miR-489 | 86 |
| hsa-miR-499a-3p | 90 |
| hsa-miR-627 | 109 |
| hsa-miR-302d-3p | 142 |
| hsa-miR-631 | 147 |
| hsa-miR-574-5p | 148 |
| hsa-miR-1283 | 217 |
| hsa-miR-93-5p | 279 |
| hsa-miR-4454 | 395 |
| hsa-miR-1246 | 657 |
| hsa-miR-212-3p | 809 |

Supplementary Table S2: List of Differentially expressed microRNAs in p53/exosomes as compared to SK/exo

| p53/ exo increased | Nanostring counts |
| --- | --- |
| hsa-miR-130a-3p | 8059 |
| hsa-miR-193a-5p | 3649 |
| hsa-miR-578 | 1058 |
| hsa-miR-379-5p | 1029 |
| hsa-miR-15a-5p | 1018 |
| hsa-miR-100-5p | 657 |
| hsa-miR-505-3p | 421 |
| hsa-miR-382-5p | 395 |
| hsa-miR-889 | 279 |
| hsa-miR-501-3p | 159 |
