# Supplementary material for: Modification of tumor cell exosome content by transfection with wt-p53 and microRNA-125b expressing plasmid DNA and its effect on macrophage polarization
Source: Oncogenesis. 2016 Aug 8;5(8):e250–. doi: 10.1038/oncsis.2016.52 (PMC5007827; doi:10.1038/oncsis.2016.52)
Supplement: Supplementary Table 3 [file oncsis201652x13.docx]

**Supplementary Table S3: List of Differentially expressed microRNAs in 125b/exosomes as compared to SK/exo**

| **125 decreased** | **Nanostring Counts** |  |  | **125 increased** | **Nanostring Counts** |
| --- | --- | --- | --- | --- | --- |
| hsa-miR-410 | 38 |  |  | hsa-miR-125b-5p | 100738 |
| hsa-miR-576-5p | 40 |  |  | hsa-let-7a-5p | 10029 |
| hsa-miR-196a-5p | 46 |  |  | hsa-let-7i-5p | 5408 |
| hsa-miR-337-5p | 47 |  |  | hsa-miR-210 | 3855 |
| hsa-miR-154-5p | 49 |  |  | hsa-miR-486-3p | 3002 |
| hsa-miR-1915-3p | 51 |  |  | hsa-miR-429 | 2338 |
| hsa-miR-30a-5p | 51 |  |  | hsa-miR-182 | 1605 |
| hsa-miR-32-5p | 51 |  |  | hsa-miR-126-3p | 1511 |
| hsa-miR-590-5p | 52 |  |  | hsa-miR-34b-3p | 1196 |
| hsa-miR-145-5p | 54 |  |  | hsa-miR-191-5p | 1058 |
| hsa-miR-30e-5p | 54 |  |  | hsa-let-7b-5p | 1041 |
| hsa-miR-489 | 55 |  |  | hsa-miR-1248 | 1019 |
| hsa-miR-19a-3p | 56 |  |  | hsa-miR-133a | 1011 |
| hsa-miR-323a-3p | 56 |  |  | hsa-miR-632 | 871 |
| hsa-miR-630 | 56 |  |  | hsa-miR-146a-5p | 818 |
| hsa-miR-19b-3p | 57 |  |  | hsa-miR-181a-5p | 805 |
| hsa-miR-338-3p | 58 |  |  | hsa-miR-29b-3p | 799 |
| hsa-miR-148b-3p | 59 |  |  | hsa-miR-320e | 755 |
| hsa-miR-370 | 59 |  |  | hsa-miR-23a-3p | 465 |
| hsa-miR-411-5p | 61 |  |  | hsa-let-7g-5p | 455 |
| hsa-miR-198 | 62 |  |  | hsa-miR-197-3p | 400 |
| hsa-miR-30b-5p | 63 |  |  | hsa-miR-125a-3p | 394 |
| hsa-miR-188-5p | 66 |  |  | hsa-miR-1290 | 286 |
| hsa-miR-518a-3p | 69 |  |  | hsa-miR-187-3p | 283 |
| hsa-miR-598 | 69 |  |  | hsa-miR-151a-5p | 259 |
| hsa-miR-631 | 74 |  |  | hsa-miR-130a-3p | 223 |
| hsa-miR-93-5p | 76 |  |  | hsa-miR-29a-3p | 213 |
| hsa-miR-601 | 82 |  |  | hsa-miR-720 | 198 |
| hsa-miR-10a-5p | 86 |  |  | hsa-miR-106b-5p | 191 |
| hsa-miR-1225-5p | 96 |  |  | hsa-miR-431-5p | 179 |
| hsa-miR-378e | 102 |  |  | hsa-miR-451a | 174 |
| hsa-miR-574-5p | 115 |  |  | hsa-miR-15a-5p | 159 |
| hsa-miR-514b-5p | 154 |  |  | hsa-miR-541-3p | 157 |
| hsa-miR-25-3p | 238 |  |  | hsa-miR-16-5p | 156 |
| hsa-miR-302d-3p | 241 |  |  | hsa-miR-183-5p | 152 |
| hsa-miR-144-3p | 275 |  |  | hsa-miR-100-5p | 150 |
| hsa-miR-135b-5p | 409 |  |  | hsa-miR-34a-5p | 141 |
| hsa-miR-1283 | 548 |  |  | hsa-miR-30d-5p | 123 |
| hsa-miR-222-3p | 605 |  |  | hsa-miR-374a-5p | 93 |
| hsa-miR-1246 | 855 |  |  | hsa-miR-15b-5p | 81 |
| hsa-miR-212-3p | 1047 |  |  | hsa-miR-1972 | 79 |
| hsa-miR-4454 | 1590 |  |  | hsa-miR-888-5p | 78 |
|  |  |  |  | hsa-miR-330-3p | 65 |
|  |  |  |  | hsa-miR-95 | 60 |
|  |  |  |  | hsa-miR-378g | 56 |
|  |  |  |  | hsa-miR-936 | 56 |
|  |  |  |  | hsa-miR-192-5p | 54 |
|  |  |  |  | hsa-miR-409-5p | 51 |
