# Supplementary material for: Modification of tumor cell exosome content by transfection with wt-p53 and microRNA-125b expressing plasmid DNA and its effect on macrophage polarization
Source: Oncogenesis. 2016 Aug 8;5(8):e250–. doi: 10.1038/oncsis.2016.52 (PMC5007827; doi:10.1038/oncsis.2016.52)
Supplement: Supplementary Table 4 [file oncsis201652x14.docx]

**Supplementary Table S4: List of Differentially expressed microRNAs in combi/exosomes as compared to SK/exo**

| **Combi/exo decreased** | **Nanostring Counts** |  | **combi/exo increased** | **Nanostring Counts** |
| --- | --- | --- | --- | --- |
| hsa-miR-34a-5p | 36 |  | hsa-miR-191-5p | 59665 |
| hsa-miR-335-5p | 42 |  | hsa-miR-16-5p | 47791 |
| hsa-miR-15b-5p | 43 |  | hsa-miR-29b-3p | 34933 |
| hsa-miR-23b-3p | 44 |  | hsa-miR-130a-3p | 32394 |
| hsa-miR-198 | 49 |  | hsa-let-7a-5p | 30072 |
| hsa-miR-423-3p | 49 |  | hsa-miR-148b-3p | 19037 |
| hsa-miR-449b-5p | 49 |  | hsa-miR-206 | 18520 |
| hsa-miR-15a-5p | 51 |  | hsa-miR-210 | 15380 |
| hsa-miR-486-3p | 51 |  | hsa-miR-19b-3p | 13871 |
| hsa-miR-2682-5p | 52 |  | hsa-miR-125b-5p | 13382 |
| hsa-miR-1224-5p | 54 |  | hsa-miR-25-3p | 12803 |
| hsa-miR-136-5p | 58 |  | hsa-miR-146a-5p | 10248 |
| hsa-miR-1246 | 59 |  | hsa-miR-30b-5p | 9157 |
| hsa-miR-544b | 65 |  | hsa-miR-100-5p | 7885 |
| hsa-miR-129-2-3p | 75 |  | hsa-miR-196a-5p | 7276 |
| hsa-miR-378b | 82 |  | hsa-miR-28-3p | 6615 |
| hsa-miR-631 | 127 |  | hsa-miR-338-3p | 6175 |
|  |  |  | hsa-miR-106b-5p | 6074 |
|  |  |  | hsa-miR-19a-3p | 5487 |
|  |  |  | hsa-miR-342-5p | 5238 |
|  |  |  | hsa-miR-181a-5p | 5112 |
|  |  |  | hsa-miR-1290 | 4046 |
|  |  |  | hsa-miR-4286 | 4042 |
|  |  |  | hsa-miR-378g | 3960 |
|  |  |  | hsa-miR-379-5p | 3777 |
|  |  |  | hsa-miR-431-5p | 3660 |
|  |  |  | hsa-miR-222-3p | 3586 |
|  |  |  | hsa-miR-4458 | 3275 |
|  |  |  | hsa-miR-337-5p | 3263 |
|  |  |  | hsa-let-7g-5p | 3233 |
|  |  |  | hsa-let-7i-5p | 2974 |
|  |  |  | hsa-miR-127-3p | 2485 |
|  |  |  | hsa-miR-497-5p | 2425 |
|  |  |  | hsa-miR-323a-3p | 2209 |
|  |  |  | hsa-miR-384 | 2164 |
|  |  |  | **combi/exo increased** | **Nanostring Counts** |
|  |  |  | hsa-miR-10a-5p | 2055 |
|  |  |  | hsa-miR-4284 | 1844 |
|  |  |  | hsa-miR-130b-3p | 1760 |
|  |  |  | hsa-miR-34b-3p | 1688 |
|  |  |  | hsa-miR-365a-3p | 1458 |
|  |  |  | hsa-miR-148a-3p | 1380 |
|  |  |  | hsa-miR-325 | 1318 |
|  |  |  | hsa-miR-126-3p | 1275 |
|  |  |  | hsa-miR-122-5p | 1263 |
|  |  |  | hsa-miR-154-5p | 1088 |
|  |  |  | hsa-miR-133a | 1081 |
|  |  |  | hsa-miR-33a-5p | 1023 |
|  |  |  | hsa-miR-330-3p | 935 |
|  |  |  | hsa-miR-1 | 928 |
|  |  |  | hsa-miR-374c-5p | 896 |
|  |  |  | hsa-let-7b-5p | 653 |
|  |  |  | hsa-miR-410 | 633 |
|  |  |  | hsa-miR-192-5p | 604 |
|  |  |  | hsa-miR-128 | 465 |
|  |  |  | hsa-miR-499a-3p | 459 |
|  |  |  | hsa-miR-505-3p | 458 |
|  |  |  | hsa-miR-183-5p | 424 |
|  |  |  | hsa-miR-4421 | 362 |
|  |  |  | hsa-let-7f-5p | 350 |
|  |  |  | hsa-miR-411-5p | 317 |
|  |  |  | hsa-miR-151a-5p | 289 |
|  |  |  | hsa-miR-487b | 284 |
|  |  |  | hsa-miR-29a-3p | 197 |
|  |  |  | hsa-miR-425-5p | 87 |
